# Finerenone in People with CKD, Type 2 Diabetes, and History of Nephrectomy

**DOI:** 10.2215/CJN.0000000932

**Published:** 2026-01-14

**Authors:** Jair Munoz Mendoza, Matthew R. Weir, Stefan D. Anker, Gerasimos Filippatos, Peter Rossing, Christiane Ahlers, Meike Brinker, Samuel T. Fatoba, Andrea Horvat-Broecker, Katja Rohwedder, Alessia Fornoni

**Affiliations:** 1 Katz Family Division of Nephrology and Hypertension, Department of Medicine, Peggy and Harold Katz Family Drug Discovery Center, University of Miami, Miller School of Medicine, Miami, Florida; 2 Department of Medicine, University of Maryland School of Medicine, Baltimore, Maryland; 3 Department of Cardiology (CVK) of German Heart Center Charité, German Centre for Cardiovascular Research (DZHK) Partner Site Berlin, Charité Universitätsmedizin, Berlin, Germany; 4 Department of Cardiology, School of Medicine, Attikon University Hospital, National and Kapodistrian University of Athens, Athens, Greece; 5 Steno Diabetes Center Copenhagen, Copenhagen, Denmark; 6 Department of Clinical Medicine, University of Copenhagen, Copenhagen, Denmark; 7 Statistics and Data Insights, Bayer AG, Wuppertal, Germany; 8 Cardiology and Nephrology Clinical Development, Bayer AG, Wuppertal, Germany; 9 Bayer US, LLC, Medical Affairs, Whippany, New Jersey; 10 Bayer AG, Medical Affairs and Pharmacovigilance, Wuppertal, Germany; 11 Cardio-Renal Medical Affairs Department, Bayer AG, Berlin, Germany

**Keywords:** CKD, chronic kidney failure, diabetes, kidney disease, kidney dysfunction, nephrectomy, progression of renal failure, diabetic kidney disease

## Abstract

**Key Points:**

- Finerenone reduced albuminuria versus placebo in patients with a history of nephrectomy, similar to those without a history of nephrectomy.
- Incidences of treatment-emergent adverse events or serious adverse events were generally similar in patients with and without history of nephrectomy.
- Finerenone may delay kidney disease progression in patients with CKD and type 2 diabetes, irrespective of nephrectomy status.

**Background:**

Finerenone significantly reduced the risk of cardiovascular and kidney outcomes in patients with CKD and type 2 diabetes (T2D) in FIDELITY, a prespecified pooled analysis of two phase 3 trials. This *post hoc* FIDELITY analysis examined the efficacy and safety of finerenone in patients with CKD, T2D, and a history of nephrectomy.

**Methods:**

Patients in FIDELITY were randomized to receive finerenone or placebo and were on optimized renin–angiotensin system inhibition. We identified nephrectomy status using patients' medical history and assessed CKD progression in patients by nephrectomy status at baseline by modeling change in urine albumin-to-creatinine ratio from baseline to months 4–24. Safety outcomes included treatment-emergent adverse events and incident hyperkalemia.

**Results:**

Of 12,990 patients, 108 had a history of nephrectomy at baseline; 101 of 108 had radical nephrectomy, 55 received finerenone, and 53 received placebo. Baseline mean eGFR were numerically lower in patients with a history of nephrectomy (48±17 ml/min per 1.73 m^2^) than in patients without (58±22 ml/min per 1.73 m^2^). For patients with a history of nephrectomy, those who received finerenone had a greater urine albumin-to-creatinine ratio reduction at 4 months versus those who received placebo (least-squares mean ratio to baseline, 0.65 versus 1.09; least-squares mean treatment ratio, 0.60; 95% confidence interval, 0.48 to 0.76; *P* < 0.001). This reduction was maintained for 2 years. Treatment-emergent adverse events were similar in patients with and without a history of nephrectomy. Among patients with a history of nephrectomy, treatment-emergent hyperkalemia occurred in 7% and 6% of finerenone and placebo groups, respectively.

**Conclusions:**

Finerenone reduced albuminuria compared with placebo and demonstrated a safety profile consistent with the overall FIDELITY population in patients with and without a history of nephrectomy at baseline. Finerenone may delay CKD progression and associated morbidity in patients with CKD and T2D, irrespective of nephrectomy status.

**Clinical Trial registry name and registration number:**

FIDELIO-DKD (NCT02540993); FIGARO-DKD (NCT02545049).

## Visual Abstract

**Figure:**
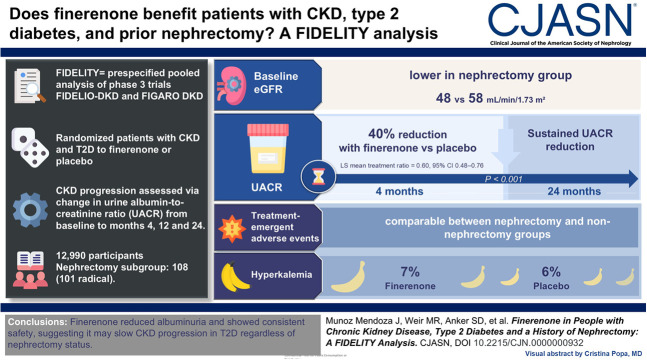

## Introduction

Nephrectomy is the treatment of choice for localized kidney tumors^1^ and for some cases of severe nephrolithiasis and pyelonephritis^2^; it is also performed for living kidney donation. In patients with CKD caused by T2D, hypertension, or GN, kidney function usually declines with the progression of the underlying cause.^3–5^ The influence of nephrectomy on CKD in these patients is not well understood. Evidence suggests that nephrectomy is associated with higher risk of CKD, kidney failure, and cardiovascular (CV) events.^6–10^ Risk factors for CKD after nephrectomy include older age, history of CKD, lower eGFR and higher urine albumin-to-creatinine ratio (UACR) pre-nephrectomy, T2D, and a radical rather than partial nephrectomy.^7,8,10–12^ However, individuals who have CKD at the time of nephrectomy may experience more rapid CKD progression than individuals who develop *de novo* CKD after nephrectomy.^3,13^ CKD and albuminuria are frequently present in patients who undergo nephrectomy,^14^ and the incidence of albuminuria seems to increase postprocedure.^15^ Consequently, effective therapies are needed for this at-risk population.

Finerenone, a nonsteroidal mineralocorticoid receptor antagonist, significantly reduced the risk of adverse CV and kidney outcomes in patients with CKD and T2D in FIDELITY, a prespecified pooled analysis of the phase 3 FIDELIO-DKD (Finerenone in Reducing Kidney Failure and Disease Progression in Diabetic Kidney Disease; ClinicalTrials.gov ID: NCT02540993) and FIGARO-DKD (Finerenone in Reducing CV Mortality and Morbidity in Diabetic Kidney Disease; ClinicalTrials.gov ID: NCT02545049) trials.^16^ In these trials, finerenone also reduced UACR by 32% compared with placebo after 4 months, an effect that was maintained for 2 years.^16^

The Kidney Disease: Improving Global Outcomes guidelines recommend measuring albuminuria for CKD risk classification and management.^17^ Albuminuria is also increasingly recognized as a biomarker for CV disease (CVD)^18^ and has been associated with incident coronary artery disease and CV mortality,^19^ stroke,^20^ and heart failure.^21^ Albuminuria is frequently present in patients undergoing nephrectomy^14,22,23^ and has been associated with greater risks of progressive CKD and mortality after both partial and radical nephrectomy.^14^ Consequently, the American Urological Association guidelines recommend assessing proteinuria and CKD stage in patients with kidney tumors who may be eligible for nephrectomy.^24^ Although data on postnephrectomy albuminuria in humans are scarce, animal studies have shown increases in aldosterone and albuminuria after nephron mass reduction following renal ablation,^25^ which are changes likely to involve disruption of the renin–angiotensin system (RAS)^26^ and the development of hyperfiltration associated with nephron loss.^27^

Data on the risk of CKD progression with finerenone in patients with CKD, T2D, and a history of nephrectomy have not been previously published. Therefore, this *post hoc* analysis examined the efficacy and safety of finerenone in patients with CKD and T2D from FIDELITY who had undergone nephrectomy, using UACR as a surrogate for CKD progression.

## Methods

### Study Design and Participants

FIDELITY combined individual patient-level data from FIDELIO-DKD (ClinicalTrials.gov ID: NCT02540993) and FIGARO-DKD (ClinicalTrials.gov ID: NCT02545049), two phase 3, randomized, double-blind, placebo-controlled trials. The study designs of these trials and the primary results of the FIDELITY analysis have been published previously.^16,28,29^ In brief, adults aged 18 years or older with CKD (defined as UACR 30 mg/g to <300 mg/g with eGFR 25 ml/min per 1.73 m^2^ to <60 ml/min per 1.73 m^2^ and diabetic retinopathy or UACR 300–5000 mg/g and eGFR 25 ml/min per 1.73 m^2^ to <75 ml/min per 1.73 m^2^ in FIDELIO-DKD and UACR 30 mg/g to <300 mg/g with eGFR of 25–90 ml/min per 1.73 m^2^ or UACR 300–5000 mg/g and eGFR 60 ml/min per 1.73 m^2^ or higher in FIGARO-DKD) and T2D on maximum tolerated doses of RAS inhibitors were eligible. Patients with nondiabetic kidney disease, recent history of dialysis for acute kidney failure or kidney transplant, uncontrolled hypertension, symptomatic chronic heart failure with reduced ejection fraction, or decompensated hepatic impairment (Child-Pugh class C) were excluded. Patients were randomly assigned 1:1 to treatment with finerenone (10 mg or 20 mg) or matching placebo. The starting dose for finerenone was 10 mg/d if eGFR was 25 ml/min per 1.73 m^2^ to <60 ml/min per 1.73 m^2^ and 20 mg/d if eGFR was 60 ml/min per 1.73 m^2^ or higher. Up-titration to 20 mg/d was permitted at or any time after month 1 and down-titration to 10 mg/d at any time after the start of treatment. The trials were conducted in accordance with the principles of the Declaration of Helsinki, and the protocols were approved by relevant regulatory authorities and ethics committees for each trial site. All participants provided written informed consent.

### Assessment of Nephrectomy History

Nephrectomy was identified in patients' medical history on the basis of Medical Dictionary for Regulatory Activities Preferred Term, and patients were grouped on the basis of history of nephrectomy (yes versus no). The following were recorded for patients with nephrectomy: location (right versus left), type (partial versus radical, with radical nephrectomy assumed if type was not available in the patient's history), and procedure date.

### Outcomes

The efficacy outcome for this *post hoc* analysis was change in UACR, assessed as a surrogate marker of CKD progression. In the FIDELIO-DKD and FIGARO-DKD trials, central laboratory measurements of UACR were conducted at baseline, month 4, month 12, and annually thereafter, using the first morning void urine samples collected at the patient's home on three consecutive days. In this analysis, changes from baseline to months 4, 12, and 24 were examined. Safety assessments included investigator-reported treatment-emergent adverse events (AEs [TEAEs]; defined as AEs that started or worsened after the first dose of study drug, up to 3 days after any temporary or permanent interruption of study drug) and hyperkalemia events, as well as events leading to treatment discontinuation and hospitalization. Central laboratory evaluations of hyperkalemia (serum potassium levels over 5.5 and over 6.0 mmol/L) were also assessed. Efficacy (UACR change) and safety (overall TEAEs and hyperkalemia) outcomes were assessed for finerenone versus placebo by history of nephrectomy at baseline.

### Statistical Analysis

Baseline characteristics and efficacy outcomes were reported for the full analysis set, which included all randomized participants who did not have critical Good Clinical Practice violations. Safety analyses were performed on the safety analysis set, which included randomized participants without critical Good Clinical Practice violations who took at least one dose of study drug. Ratio to baseline UACR was analyzed for each subgroup category using a mixed model with factors treatment group, region, eGFR category at screening, type of albuminuria at screening, CVD history, study, time, treatment by time, baseline value nested within eGFR category at screening, and baseline value by time and treatment by study interactions as covariates. Baseline value by time was included as a covariate in this model because it was expected that the influence of baseline values on UACR would vary across time points. Given that raw UACR values exhibited a high degree of positive skewness (2.0), log-transformed values were used in the models to normalize their distribution. A UACR analysis in an unadjusted model was also performed for comparison. An analysis of covariance was also performed with factors treatment group, region, eGFR category at screening, type of albuminuria at screening, CVD history, study, log-transformed baseline value nested within type of albuminuria, and interaction between study and treatment. All analyses were performed using SAS software, version 9.4 (SAS Institute, Cary, NC).

## Results

### Patients

Of 12,990 patients included in the FIDELITY full analysis set, 108 (0.8%) had a history of nephrectomy at baseline, with 101 (94%) having undergone radical nephrectomy. In the nephrectomy group, 55 patients received finerenone and 53 patients received placebo. The mean finerenone treatment duration was 32.5 months (SD 12.3) in patients with a history of nephrectomy and 31.6 months (SD 14.4) in patients without. The median time from nephrectomy to randomization was approximately 15 years (Supplemental Figure 1).

### Baseline Characteristics

Among patients with a history of nephrectomy, the mean age was 66 years, and 61% were male (Table 1). For finerenone-treated patients, median (interquartile range) UACR at baseline was 670.8 (209.6–1098.5) mg/g in patients with a history of nephrectomy and 513.9 (197.2–1132.9) mg/g in patients without a history of nephrectomy. Respective values in the placebo groups were 503.8 (216.8–1115.6) mg/g and 515.5 (199.0–1165.7) mg/g. Almost 100% of patients across treatment arms in both groups were treated with angiotensin-converting enzyme inhibitors or angiotensin receptor blockers at baseline.

**Table 1:** Baseline demographics and clinical characteristics according to nephrectomy status in medical history (full analysis set)

Characteristic	Nephrectomy (*n*=108)			No Nephrectomy (*n*=12,882)		
	Finerenone (*n*=55)	Placebo (*n*=53)	Total (*n*=108)	Finerenone (*n*=6443)	Placebo (*n*=6439)	Total (*n*=12,882)
Age, yr, mean±SD	65.6±9.6	66.3±8.5	65.9±9.0	64.7±9.4	64.8±9.7	64.8±9.5
Sex, male, *n* (%)	34 (62)	32 (60)	66 (61)	4429 (69)	4563 (71)	8992 (70)
**Race, *n* (%)**						
Asian	3 (5)	3 (6)	6 (6)	1410 (22)	1444 (22)	2854 (22)
Black/African American	1 (2)	1 (2)	2 (2)	250 (4)	268 (4)	518 (4)
White	51 (93)	48 (91)	99 (92)	4398 (68)	4372 (68)	8770 (68)
Systolic BP, mm Hg, mean±SD	137.5±13.1	136.9±12.3	137.2±12.7	136.8±14.2	136.7±14.3	136.8±14.2
Diastolic BP, mm Hg, mean±SD	78.6±8.6	76.4±9.6	77.5±9.1	76.3±9.6	76.4±9.6	76.3±9.6
BMI, kg/m^2^, mean±SD	33.2±6.6	32.3±5.4	32.8±6.1	31.3±6.0	31.3±6.0	31.3±6.0
Duration of diabetes, yr, mean±SD	13.3±7.2	14.3±9.3	13.8±8.3	15.5±8.7	15.4±8.7	15.4±8.7
HbA1c, %, mean±SD	7.5±1.2	7.5±1.5	7.5±1.4	7.7±1.4	7.7±1.4	7.7±1.4
Serum potassium, mmol/L, mean±SD	4.40±0.4	4.36±0.5	4.4±0.5	4.4±0.4	4.4±0.4	4.4±0.4
eGFR,^a^ ml/min per 1.73 m^2^, mean±SD	48±16	47±18	48±17	58±22	58±22	58±22
**eGFR,**^a^ **ml/min per 1.73 m**^**2**^**, *n* (%)**						
<25	1 (2)	3 (6)	4 (4)	80 (1)	78 (1)	158 (1)
25–<45	25 (45)	26 (49)	51 (47)	2087 (32)	2086 (32)	4173 (32)
45–<60	15 (27)	11 (21)	26 (24)	1696 (26)	1704 (26)	3400 (26)
≥60	14 (25)	13 (25)	27 (25)	2579 (40)	2569 (40)	5148 (40)
UACR, mg/g, median (Q1–Q3)	670.8 (209.6–1098.5)	503.8 (216.8–1115.6)	538.9 (209.6–1115.3)	513.9 (197.2–1132.9)	515.5 (199.0–1165.7)	514.7 (197.9–1149.1)
**UACR, mg/g, *n* (%)**						
<30	0	0	0	120 (2)	110 (2)	230 (2)
30–<300	17 (31)	16 (30)	33 (31)	2049 (32)	1999 (31)	4048 (31)
≥300	37 (67)	37 (70)	74 (69)	4273 (66)	4327 (67)	8600 (67)
Waist–hip ratio, mean±SD	1.0±0.1	1.00±0.1	1.0±0.1	1.0±0.1	1.0±0.1	1.0±0.1
Hip circumference, cm, mean±SD	111.9±16.0	110.2±12.1	111.1±14.2	107.5±14.0	107.5±13.7	107.5±13.9
Waist circumference, cm, mean±SD	111.1±16.5	109.8±13.2	110.4±14.9	107.0±15.1	107.1±15.1	107.0±15.1
hs-CRP, mg/dl, mean±SD	6.5±13.3	4.5±4.4	5.5±10.0	4.8±10.4	4.7±9.2	4.7±9.9
Heart rate, bpm, mean±SD	71.4±11.2	72.8±13.0	72.1±12.0	73.2±11.4	73.0±11.4	73.0±11.4
Current smoker, *n* (%)	7 (13)	8 (15)	15 (14)	1052 (16)	1015 (16)	2067 (16)
**Medical history findings, *n* (%)**						
Hyperkalemia	2 (4)	1 (2)	3 (3)	110 (2)	111 (2)	221 (2)
Hypertension	53 (96)	53 (100)	106 (98)	6207 (96)	6218 (97)	12,425 (96)
Hyperlipidemia	25 (45)	28 (53)	53 (49)	2770 (43)	2802 (44)	5572 (44)
Diabetic retinopathy	10 (18)	15 (28)	25 (23)	2488 (39)	2428 (38)	4916 (38)
CAD	14 (25)	18 (34)	32 (30)	1973 (31)	1988 (31)	3961 (31)
Diabetic neuropathy	14 (25)	15 (28)	29 (27)	1772 (28)	1697 (26)	3469 (27)
Peripheral arterial occlusive disease	9 (16)	6 (11)	15 (14)	1045 (16)	1021 (16)	2066 (16)
MI	8 (15)	8 (15)	16 (15)	1008 (16)	996 (15)	2004 (16)
Ischemic stroke	6 (11)	7 (13)	13 (12)	763 (12)	778 (12)	1541 (12)
Heart failure	6 (11)	4 (8)	10 (9)	479 (7)	518 (8)	997 (8)
AFF	4 (7)	0	4 (4)	562 (9)	538 (8)	1100 (9)
**Medication use at baseline,**^b^ ***n* (%)**						
RASi	55 (100)	53 (100)	108 (100)	6432 (100)	6427 (100)	12,859 (100)
*β*-blockers	31 (56)	32 (60)	63 (58)	3201 (50)	3235 (50)	6436 (50)
Diuretics	33 (60)	33 (62)	66 (61)	3285 (51)	3350 (52)	6635 (52)
*Loop diuretics*	17 (31)	13 (25)	30 (28)	1362 (21)	1408 (22)	2770 (22)
*Thiazide diuretics*	16 (29)	17 (32)	33 (31)	1589 (25)	1524 (24)	3113 (24)
Statins	41 (75)	38 (72)	79 (73)	4610 (72)	4698 (73)	9308 (72)
Potassium supplements	2 (4)	4 (8)	6 (6)	194 (3)	185 (3)	379 (3)
Potassium-lowering agents	3 (5)	1 (2)	4 (4)	91 (1)	87 (1)	178 (1)
**Antidiabetic medications**						
Insulins and analogues	27 (49)	32 (60)	59 (55)	3831 (59)	3730 (60)	7561 (59)
DPP-4 inhibitors	14 (25)	15 (28)	29 (27)	1632 (25)	1594 (25)	3226 (25)
GLP-1RAs	6 (11)	4 (8)	10 (9)	491 (8)	442 (7)	933 (7)
SGLT-2 inhibitors	3 (5)	2 (4)	5 (5)	433 (7)	435 (7)	868 (7)
Metformin	27 (49)	22 (42)	49 (45)	3774 (59)	3716 (58)	7490 (58)
Sulfonamides	15 (27)	10 (19)	25 (23)	1671 (26)	1682 (26)	3353 (26)
*α*-glucosidase inhibitors	1 (2)	2 (4)	3 (3)	321 (5)	328 (5)	649 (5)
Meglitinides	3 (5)	1 (2)	4 (4)	269 (4)	256 (4)	525 (4)
Thiazolidinediones	0	2 (4)	2 (2)	267 (4)	246 (4)	513 (4)

AFF, atrial fibrillation and atrial flutter; BMI, body mass index; bpm, beats per minute; CAD, coronary artery disease; DDP-4, dipeptidyl peptidase-4; GLP-1RA, glucagon-like peptide-1 receptor agonist; HbA1c, hemoglobin A1c; hs-CRP, high-sensitivity C-reactive protein; MDRD, modification of diet in renal disease; MI, myocardial infarction; Q, quartile; RASi, renin–angiotensin system inhibitor; SGLT-2, sodium-glucose co-transporter-2; UACR, urine albumin-to-creatinine ratio.

a eGFR was measured using the modification of diet in renal disease formula with risk factor included.

b Multiple drug groups per drug are possible; the same drug may be counted in more than one category for the same patient.

Generally, baseline characteristics were similar between treatment arms within the two groups. However, some baseline differences were reported between patients with and without a history of nephrectomy (Table 1). Compared with patients without a history of nephrectomy, more patients with a history of nephrectomy were White (68% versus 92%, respectively) and fewer were of Asian race (22% versus 6%, respectively). The mean eGFR was lower in patients with a history of nephrectomy (48±17 ml/min per 1.73 m^2^) than in patients without a history of nephrectomy (58±22 ml/min per 1.73 m^2^), and a greater proportion of patients with a history of nephrectomy had an eGFR <45 ml/min per 1.73 m^2^ (51% versus 34% without). Patients with a history of nephrectomy tended to have a slightly shorter duration of diabetes than those without a history of nephrectomy (13.8±8.3 versus 15.4±8.7 years, respectively), and fewer had a history of diabetic retinopathy (23% versus 38%, respectively); fewer patients with a history of nephrectomy were on sodium-glucose co-transporter-2 inhibitors (5% versus 7% of those without) or metformin (45% versus 58% of those without). In addition, a medical history of atrial fibrillation/flutter (4% versus 9%) and peripheral arterial occlusive disease (14% versus 16%) was less common, whereas heart failure was more common (9% versus 8%) in patients with a history of nephrectomy compared with those without, respectively. Otherwise, baseline characteristics were similar between the nephrectomy and no-nephrectomy groups (Table 1).

### Efficacy: Change in UACR from Baseline

**Figure 1:**
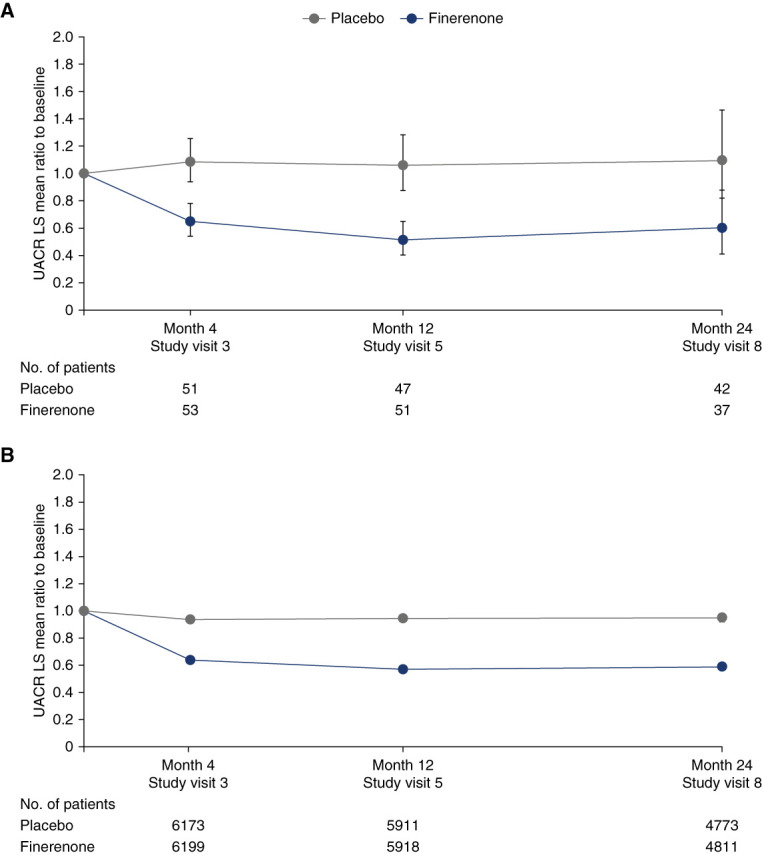
**Changes in UACR from baseline.** (A) Patients with a history of nephrectomy. (B) Patients without a history of nephrectomy. LS mean and 95% CI results from a mixed model with separate unstructured covariance patterns for each treatment group, with factors including treatment group, region, eGFR category at screening, type of albuminuria at screening, CVD history, study, time, interaction between treatment and time, baseline value nested within eGFR category at screening, and interaction between baseline value and time as covariate. CI, confidence interval; CVD, cardiovascular disease; LS, least-squares; UACR, urine albumin-to-creatinine ratio.

Treatment with finerenone reduced UACR from baseline to month 4 compared with placebo in patients with and without a history of nephrectomy by 40% and 32%, respectively. For patients with a history of nephrectomy, the least-squares (LS) mean ratios to baseline at 4 months for finerenone and placebo were 0.65 and 1.09 (LS mean treatment ratio, 0.60; 95% confidence interval, 0.48 to 0.76; *P* < 0.001; Figure 1A and Table 2). For patients without a history of nephrectomy, the respective LS mean ratios to baseline were 0.64 and 0.94 (LS mean treatment ratio, 0.68; 95% confidence interval, 0.66 to 0.70; *P* < 0.001; Figure 1B and Table 2). Nominally significant reductions in UACR with finerenone versus placebo were maintained up to 24 months in the subgroups with and without a history of nephrectomy (Figure 1, A and B and Table 2), and there was no significant heterogeneity in treatment effects between the studies (*P*_interaction_ values between treatment [finerenone or placebo] and study [FIDELIO-DKD or FIGARO-DKD] were 0.61 and 0.80 for the with and without nephrectomy subgroups, respectively; Supplemental Table 1). Factors including region, albuminuria level at screening, eGFR category, history of CVD at baseline, and study (FIDELIO-DKD or FIGARO-DKD) did not significantly influence UACR change at month 4, among all patients with a history of nephrectomy (Supplemental Table 2). Results of the unadjusted UACR ratio to baseline analysis were consistent with the adjusted UACR reduction analysis for patients with and without a history of nephrectomy (Supplemental Table 3).

**Table 2:** Urine albumin-to-creatinine ratio reductions up to 2 years according to nephrectomy status in medical history (full analysis set)

Visit	Nephrectomy				No Nephrectomy			
	Treatment (*n*/*N*)	LS Mean Ratio to Baseline (95% CI)	LS Mean Treatment Ratio (95% CI)	*P* Value	Treatment (*n*/*N*)	LS Mean Ratio to Baseline (95% CI)	LS Mean Treatment Ratio (95% CI)	*P* Value
Month 4	Finerenone (53/55)	0.65 (0.54 to 0.78)	0.60 (0.48 to 0.76)	<0.001	Finerenone (6199/6443)	0.64 (0.62 to 0.65)	0.68 (0.66 to 0.70)	<0.001
	Placebo (51/53)	1.07 (0.94 to 1.26)			Placebo (6173/6439)	0.94 (0.92 to 0.95)		
Month 12	Finerenone (51/55)	0.51 (0.40 to 0.65)	0.48 (0.36 to 0.65)	<0.001	Finerenone (5918/6443)	0.57 (0.55 to 0.59)	0.60 (0.58 to 0.63)	<0.001
	Placebo (47/53)	1.06 (0.88 to 1.28)			Placebo (5911/6439)	0.94 (0.92 to 0.97)		
Month 24	Finerenone (37/55)	0.60 (0.41 to 0.88)	0.55 (0.34 to 0.88)	0.01	Finerenone (4811/6443)	0.59 (0.57 to 0.61)	0.62 (0.59 to 0.65)	<0.001
	Placebo (42/53)	1.10 (0.82 to 1.47)			Placebo (4773/6439)	0.95 (0.92 to 0.98)		

Mixed model with separate unstructured covariance patterns for each treatment group, with factors including treatment group, region, eGFR category at screening, type of albuminuria at screening, cardiovascular disease history, study, time, interaction between treatment and time, baseline value nested within eGFR category at screening, and interaction between baseline value and time and between treatment and study. CI, confidence interval; CVD, cardiovascular disease; LS, least-squares; *n*/*N*, number of patients with urine albumin-to-creatinine ratio data available for the analysis/total number of patients in subgroup.

### Safety

The incidence rates of any TEAE or treatment-emergent serious AE were generally similar in the subgroups with and without a history of nephrectomy (Table 3 and Supplemental Table 4).

**Table 3:** Treatment-emergent adverse events according to nephrectomy status in medical history (safety analysis set)

TEAE, *n* (%)	Nephrectomy		No Nephrectomy	
	Finerenone (*n*=55)	Placebo (*n*=53)	Finerenone (*n*=6434)	Placebo (*n*=6421)
Any treatment-emergent AE	44 (80)	49 (92)	5538 (86)	5543 (86)
Any treatment-emergent serious AE	19 (35)	21 (40)	2035 (32)	2160 (34)
Any treatment-emergent hyperkalemia	4 (7)	3 (6)	904 (14)	445 (7)
Leading to permanent discontinuation	0	1 (2)	110 (2)	37 (1)
Serious hyperkalemia	1 (2)	0	68 (1)	16 (0.2)
Leading to permanent discontinuation	0	0	10 (0.2)	2 (<0.1)
Leading to hospitalization	1 (2)	0	60 (0.9)	10 (0.2)
Fatal	0	0	0	0
**Central laboratory findings**				
Serum potassium over 5.5 mmol/L, *n*/*N* (%)	7/55 (13)	7/52 (13)	1065/6326 (17)	463/6303 (7)
Serum potassium over 6.0 mmol/L, *n*/*N* (%)	2/55 (4)	0/52 (0)	209/6363 (3)	80/6346 (1)

AE, adverse event; TEAE, treatment-emergent adverse events.

Treatment-emergent hyperkalemia was more common with finerenone than with placebo in patients with and without a history of nephrectomy. Among patients with a history of nephrectomy, hyperkalemia occurred in four patients (7%) treated with finerenone and three patients (6%) who received placebo; serious hyperkalemia events led to hospitalization in one patient (2%) who received finerenone (Table 3).

In patients without a history of nephrectomy, serum potassium levels over 5.5 and over 6.0 mmol/L were more frequent in patients treated with finerenone compared with patients receiving placebo (Table 3). However, levels over 5.5 mmol/L were similar in the finerenone and placebo groups in patients with a history of nephrectomy (13% versus 13%, respectively).

## Discussion

In this *post hoc* analysis of FIDELITY data, we found that compared with placebo, finerenone reduced albuminuria in patients with a history of nephrectomy, consistent with findings in patients without a history of nephrectomy. In addition, finerenone demonstrated a safety profile in patients with a history of nephrectomy that was consistent with that observed in the overall FIDELITY population.^16^ These findings support the use of finerenone to potentially improve kidney outcomes in patients with CKD, albuminuria, and T2D who have undergone nephrectomy.

Many patients who undergo nephrectomy have albuminuria,^14^ but nephrectomy also increases the risk of developing both albuminuria and CKD as well as potentially increasing the rate of CKD progression.^3,13,15^ One study by Demirjian *et al*. compared rates of CKD progression in patients who had (*1*) normal kidney function before undergoing nephrectomy (*de novo* CKD; the CKD-S group), (*2*) preexisting CKD (because of medical causes) before undergoing nephrectomy (CKD-M/S) and (*3*) CKD because of medical causes and did not require a nephrectomy (CKD-M). It found that after adjustment for factors including proteinuria, rates of CKD progression were similar in patients with CKD-M and CKD-M/S. Surgery-induced CKD without underlying medical cause had the lowest rate of progression; however, a decrease in eGFR was still observed.^3^ The study also showed that eGFR decreased postsurgery for patients in both CKD-S and CKD-M/S groups, and furthermore, patients with a postsurgery eGFR up to 40 ml/min per 1.73 m^2^ experienced increased risk of renal decline compared with patients with postsurgery eGFR >40 ml/min per 1.73 m^2^. Other studies have also identified the immediate reduction in eGFR postnephrectomy and have shown that eGFR then stabilizes, which is mirrored by an increase followed by stabilization in albuminuria.^30,31^ In this analysis, patients with a history of nephrectomy had lower eGFR at baseline compared with patients without a history of nephrectomy. Patients with higher levels of albuminuria and lower eGFR who undergo nephrectomy are at increased risk of worsening CKD because of nephron mass reduction.^14^ Compensatory glomerular hyperfiltration may help maintain kidney function in the short term, allowing eGFR to increase despite nephron loss following nephrectomy. However, among patients with either preexisting CKD or T2D, hyperfiltration in the long term may worsen kidney function, leading to increased albuminuria and progressive CKD.^32^ Finerenone treatment has been reported to result in lower incidences of CKD progression and CV events compared with placebo in high-risk patients with T2D, multiple comorbidities, albuminuria, and advanced CKD.^33^ The results of this study support the safety and effectiveness of finerenone in reducing albuminuria in this multimorbid, postnephrectomy population.

Furthermore, in animal studies, hyperaldosteronism and proteinuria have been observed after nephron mass reduction postkidney ablation, which seems to improve with renin–angiotensin–aldosterone system blockade.^25^ Albuminuria is worsened by RAS activation and reduced by RAS inhibition; however, long-term use of RAS inhibitors can lead to incomplete aldosterone suppression.^34^ Consequently, the use of nonsteroidal mineralocorticoid receptor antagonists, such as finerenone along with angiotensin-converting enzyme inhibitors or angiotensin receptor blockers is recommended for patients with CKD and T2D who have persistent albuminuria.^17^ In this study, all patients had CKD, T2D, and albuminuria, and all were on optimized RAS blockade at baseline. Finerenone reduced UACR by 40% at month 4 compared with placebo in patients who had undergone nephrectomy. A recent *post hoc* mediation analysis of FIDELITY reported that finerenone-induced reductions in UACR at month 4 mediated 84% of the improvement in long-term kidney outcomes, which included kidney failure, decrease in eGFR from baseline of at least 57%, or kidney disease death.^35^ Other studies have also found a link between albuminuria reduction and improved kidney outcomes. A meta-analysis of 21 clinical trials that mainly used RAS inhibitors reported that the risk of end-stage kidney disease was reduced by 24% for each 30% reduction in albuminuria.^36^ In recent guidelines, the American Diabetes Association recommended reducing albuminuria levels to slow CKD progression in people with diabetes and CKD who have a urinary albumin level of ≥30 mg/g, with a 30% reduction advised in patients with levels ≥300 mg/g.^37^ Notably, people with CKD and albuminuria have an increased risk of CV events.^37^

Aldosterone activation of mineralocorticoid receptors plays a key role in fibrotic processes in the kidneys.^34,38^ A recent study of nephrectomy specimens from 781 patients undergoing nephrectomy reported that each component of nephrosclerosis was independently associated with proteinuria and lower eGFR, and that greater severity of nephrosclerosis was associated with higher levels of proteinuria independently of eGFR and other clinical factors.^22^ Preclinical studies have shown that finerenone provides protection against glomerular, tubular, and vascular damage in the kidney;^39^ inhibits neointimal lesion formation after vascular injury;^40^ and prevents cardiac remodeling and fibrosis.^41^ Consequently, it is possible that finerenone may have antifibrotic effects in the remanent kidney after nephrectomy.

Increase in glomerular volume may be another mechanism for worsening kidney function after nephrectomy and the timing of RAS inhibition may play a critical role. A study in rats has shown that glomerular volume and proteinuria increase after nephrectomy, and that RAS inhibition started before nephrectomy is critical to prevent it.^42^ In addition, a study in nephrectomy specimens found that larger glomerular volume predicted progressive CKD.^43^ Whether finerenone prevents the increase of, or reduces, glomerular volume is unknown and further studies are needed to address this potential novel mechanism.

Current guidelines recommend partial nephrectomy, if feasible, rather than radical nephrectomy for individuals with kidney cancers requiring surgery.^1,24^ This minimizes nephron mass reduction and provides better preservation of kidney function compared with radical nephrectomy. In addition to radical nephrectomy, other predictors of kidney failure in patients undergoing nephrectomy include lower eGFR, higher UACR, and a history of T2D.^8^ On the basis of these predictors, an equation to calculate kidney failure risk after nephrectomy has been developed.^8^ Finerenone may reduce the risk of kidney failure in this population; however, published data are needed to confirm this.

As with the outcomes from the analyses of the overall FIDELITY population,^16^ hyperkalemia was slightly more common in finerenone-treated patients than in placebo-treated patients, irrespective of nephrectomy status. However, hyperkalemia was observed slightly less frequently in patients with a history of nephrectomy than in patients without a history of nephrectomy in both the placebo and finerenone arms. It may be difficult to draw conclusions from this observation because there were relatively few patients with a history of nephrectomy included in this analysis. The risk of hyperkalemia is known to increase during the postoperative state.^44^ The patients included in this analysis were not representative of this state given the median time from nephrectomy to randomization was 15 years, and very few patients had a history of hyperkalemia. Although the increased risk of hyperkalemia with finerenone versus placebo has been reported to be clinically manageable,^16^ serum potassium levels would need to be closely monitored if treatment were to be initiated during the perioperative phase in patients undergoing nephrectomy given the added risk of surgery-related hyperkalemia.

The exploratory nature of this analysis and the small number of patients in the FIDELIO-DKD and FIGARO-DKD trials with a history of nephrectomy at baseline relative to the overall FIDELITY population of almost 13,000 patients limit the robustness of the reported findings. In addition, the key composite CV and kidney efficacy outcomes reported in the primary FIDELITY prespecified analysis (CV: time to CV death, nonfatal myocardial infarction, nonfatal stroke, or hospitalization for heart failure; kidney: time to onset of kidney failure, sustained decrease of 57% or more in eGFR from baseline over at least 4 weeks, or kidney-related death)^16^ were not assessed in this *post hoc* analysis because the small sample size in the nephrectomy group would have resulted in a lack of power for the subgroup. This also precluded the performance of eGFR slope analyses and the extension of the current UACR analysis beyond 24 months. Evidence from previous studies supports the use of albuminuria as a surrogate endpoint for CKD progression^45–47^; thus, it was considered a suitable efficacy outcome to assess the treatment effect of finerenone in the postnephrectomy subset of the FIDELITY population. In addition, the aforementioned mediation analysis showed that a large proportion of the effect of finerenone is explained by an early reduction in UACR, further supporting the use of this outcome in the current analysis.^35^ Nevertheless, the efficacy results of this analysis should still be interpreted with caution as small numbers may increase the spread of the data.

Similarly, although the safety results of the current analysis were in line with those reported for the overall FIDELITY population,^16^ it may be difficult to draw accurate conclusions given the size of the nephrectomy group. It is worth noting that over 90% of patients in the nephrectomy group were White. Future studies would benefit from a greater representation of other racial groups to improve the generalizability of reported findings. Finally, there was a lack of information on the reason for nephrectomy in patients' medical history. Radical nephrectomy for renal cell carcinoma is associated with incident CKD and deterioration of existing CKD, whereas people with more benign conditions or living kidney donors may have less risk.^6^ Although it has been reported that albuminuria increases after living kidney donor nephrectomy, both in the short term and after long-term follow-up, this typically involves individuals with evidence of albuminuria prior to kidney donation.^30,48^

In this *post hoc* analysis, treatment with finerenone reduced albuminuria in patients with a history of nephrectomy at baseline, similarly to the cohort without a history of nephrectomy. These results suggest that finerenone may delay CKD progression and morbidity in patients with CKD and T2D irrespective of nephrectomy status and are consistent with the efficacy and safety profiles of finerenone in the overall FIDELIO-DKD and FIGARO-DKD trial populations.

## Supplementary Material

## Data Availability

Availability of the data underlying this publication will be determined according to Bayer's commitment to the EFPIA/PhRMA Principles for responsible clinical trial data sharing. This pertains to scope, timepoint, and process of data access. As such, Bayer commits to sharing upon request from qualified scientific and medical researchers' patient-level clinical trial data, study-level clinical trial data, and protocols from clinical trials in patients for medicines and indications approved in the United States and European Union as necessary for conducting legitimate research. This applies to data on new medicines and indications that have been approved by the European Union and United States regulatory agencies on or after January 01, 2014. Interested researchers can use www.vivli.org to request access to anonymized patient-level data and supporting documents from clinical studies to conduct further research that can help advance medical science or improve patient care. Information on the Bayer criteria for listing studies and other relevant information is provided in the member section of the portal. Data access will be granted to anonymized patient-level data, protocols, and clinical study reports after approval by an independent scientific review panel. Bayer is not involved in the decisions made by the independent review panel. Bayer will take all necessary measures to ensure that patient privacy is safeguarded.

## Declarative Statements

This study includes clinical experimentation and received Institutional Review Board or Ethics Committee approval. All patients provided written informed consent. This study includes clinical experimentation and complies with the Declaration of Helsinki.

## Supplemental Material

FIDELIO-DKD Investigators.

FIGARO-DKD Investigators.

Supplemental Table 1. *P* values for the main factors and interaction terms used in the mixed model for UACR ratio to baseline analysis.

Supplemental Table 2. Analysis of covariance for ratio to baseline of UACR ratio at month 4 (closest visit).

Supplemental Table 3. UACR ratio reductions up to 2 years according to nephrectomy status in medical history (unadjusted analysis; full analysis set).

Supplemental Table 4. Summary of TEAEs by body system or organ class (safety analysis set).

Supplemental Figure 1. Time from nephrectomy to randomization.
