# Supplementary material for: Finerenone in People with CKD, Type 2 Diabetes, and History of Nephrectomy
Source: Clin J Am Soc Nephrol. 2026 Jan 14;21(3):414–24. doi: 10.2215/CJN.0000000932 (PMC12959748; doi:10.2215/CJN.0000000932)
Supplement: Supplementary file 1 [file cjasn-21-414-s001.pdf]

ASN
American Society of Nephrology
JASN
Journal of the American Society of Nephrology
KIDNEY360
Accessing Our World From Every Angle
CJASN
Clinical Journal of the American Society of Nephrology

ASN Journal Disclosure Form

As per ASN journal policy, I have disclosed any financial relationships or commitments I have held in the past 36 months as included below. I have listed my Current Employer below to indicate there is a relationship requiring disclosure. If no relationship exists, my Current Employer is not listed.

C. Ahlers reports the following:
Employer: Bayer AG

I understand that the information above will be published within the journal article, if accepted, and that failure to comply and/or to accurately and completely report the potential financial conflicts of interest could lead to the following: 1) Prior to publication, article rejection, or 2) Post-publication, sanctions ranging from, but not limited to, issuing a correction, reporting the inaccurate information to the authors' institution, banning authors from submitting work to ASN journals for varying lengths of time, and/or retraction of the published work.

Name: Christiane Ahlers
Manuscript ID: CJASN-2025-000716R1
Manuscript Title: Finerenone in People with Chronic Kidney Disease, Type 2 Diabetes and a History of Nephrectomy: A FIDELITY Analysis
Date of Completion: November 10, 2025
Disclosure Updated Date: November 10, 2025

ASN American Society of Nephrology JASN Journal of the American Society of Nephrology KIDNEY360 Accessing Our World From Every Angle CJASN Clinical Journal of the American Society of Nephrology

ASN Journal Disclosure Form

As per ASN journal policy, I have disclosed any financial relationships or commitments I have held in the past 36 months as included below. I have listed my Current Employer below to indicate there is a relationship requiring disclosure. If no relationship exists, my Current Employer is not listed.

S. Anker reports the following:
Employer: Charité Medical School; Consultancy: Actimed, Alleviant, Astra Zeneca, Bayer, Berlin Heals, BiMyo, Boehringer Ingelheim, Brahms, Cardiac Dimensions, Cardior, Cordio, Corvia, CSL/Vifor, CVRx, Cytokinetics, Edwards, Impulse Dynamics, Lilly, Mankind Pharma, Novo Nordisk, Occlutech, Pfizer, Pulnovo, Regeneron, Relaxera, Repairon, Scirent, Sensible Medical, Vectorious, Veru, Viscardia, Vivus, and V-Wave.; Research Funding: Vifor Pharma, Abbott Vascular; Honoraria: Actimed, Alleviant, Astra Zeneca, Bayer, Berlin Heals, BiMyo, Boehringer Ingelheim, Brahms, Cardiac Dimensions, Cardior, Cordio, Corvia, CSL/Vifor, CVRx, Cytokinetics, Edwards, Impulse Dynamics, Lilly, Mankind Pharma, Novo Nordisk, Occlutech, Pfizer, Pulnovo, Regeneron, Relaxera, Repairon, Scirent, Sensible Medical, Vectorious, Veru, Viscardia, Vivus, and V-Wave; Patents or Royalties: Named co-inventor of two patent applications regarding MR-proANP (DE 102007010834 & DE 102007022367), but he does not benefit personally from the related issued patents.; and Advisory or Leadership Role: Actimed, Alleviant, Astra Zeneca, Bayer, Berlin Heals, Boehringer Ingelheim, Brahms, Cardiac Dimensions, Cardior, Cordio, Corvia, CVRx, Cytokinetics, Edwards, Impulse Dynamics, Lilly, Mankind Pharma, Medtronic, Novo Nordisk, Occlutech, Pfizer, Regeneron, Relaxera, Repairon, Scirent, Sensible Medical, Vectorious, Vivus, and V-Wave.

I understand that the information above will be published within the journal article, if accepted, and that failure to comply and/or to accurately and completely report the potential financial conflicts of interest could lead to the following: 1) Prior to publication, article rejection, or 2) Post-publication, sanctions ranging from, but not limited to, issuing a correction, reporting the inaccurate information to the authors' institution, banning authors from submitting work to ASN journals for varying lengths of time, and/or retraction of the published work.

Name: Stefan D. Anker
Manuscript ID: CJASN-2025-000716R2.
Manuscript Title: Finerenone in People With Chronic Kidney Disease, Type 2 Diabetes, and History of Nephrectomy.
Date of Completion: December 1, 2025
Disclosure Updated Date: December 1, 2025

ASN American Society of Nephrology
JASN Journal of the American Society of Nephrology
KIDNEY360 Accessing Our World From Every Angle
CJASN Clinical Journal of the American Society of Nephrology

ASN Journal Disclosure Form

As per ASN journal policy, I have disclosed any financial relationships or commitments I have held in the past 36 months as included below. I have listed my Current Employer below to indicate there is a relationship requiring disclosure. If no relationship exists, my Current Employer is not listed.

M. Brinker reports the following:
Employer: Bayer AG; Ownership Interest: Bayer AG; and Patents or Royalties: Description:; TREATMENT OF CHRONIC KIDNEY DISEASE IN TYPE I DIABETES MELLITUS; Patent Status:; Pending; Filing Jurisdiction:; EU; Patent Number:; WO2024/110523; Patent Holder:; Bayer.

I understand that the information above will be published within the journal article, if accepted, and that failure to comply and/or to accurately and completely report the potential financial conflicts of interest could lead to the following: 1) Prior to publication, article rejection, or 2) Post-publication, sanctions ranging from, but not limited to, issuing a correction, reporting the inaccurate information to the authors' institution, banning authors from submitting work to ASN journals for varying lengths of time, and/or retraction of the published work.

Name: Meike Daniela Brinker
Manuscript ID: CJASN-2025-000716R2
Manuscript Title: Finerenone in People With Chronic Kidney Disease, Type 2 Diabetes, and History of Nephrectomy
Date of Completion: December 1, 2025
Disclosure Updated Date: July 11, 2025

ASN American Society of Nephrology | JASN Journal of the American Society of Nephrology | KIDNEY360 Accessing Our World From Every Angle | CJASN Clinical Journal of the American Society of Nephrology

ASN Journal Disclosure Form

As per ASN journal policy, I have disclosed any financial relationships or commitments I have held in the past 36 months as included below. I have listed my Current Employer below to indicate there is a relationship requiring disclosure. If no relationship exists, my Current Employer is not listed.

S. Fatoba reports the following:
Employer: Bayer; Ownership Interest: Bayer Plc; and Other Interests or Relationships: Employee of Bayer at time of analysis.

I understand that the information above will be published within the journal article, if accepted, and that failure to comply and/or to accurately and completely report the potential financial conflicts of interest could lead to the following: 1) Prior to publication, article rejection, or 2) Post-publication, sanctions ranging from, but not limited to, issuing a correction, reporting the inaccurate information to the authors' institution, banning authors from submitting work to ASN journals for varying lengths of time, and/or retraction of the published work.

Name: Samuel T. Fatoba
Manuscript ID: CJASN-2025-000716R1
Manuscript Title: Finerenone in People with Chronic Kidney Disease, Type 2 Diabetes and a History of Nephrectomy: A FIDELITY Analysis
Date of Completion: November 20, 2025
Disclosure Updated Date: November 20, 2025

ASN American Society of Nephrology | JASN Journal of the American Society of Nephrology | KIDNEY360 Accessing Our World From Every Angle | CJASN Clinical Journal of the American Society of Nephrology

ASN Journal Disclosure Form

As per ASN journal policy, I have disclosed any financial relationships or commitments I have held in the past 36 months as included below. I have listed my Current Employer below to indicate there is a relationship requiring disclosure. If no relationship exists, my Current Employer is not listed.

G. Filippatos reports the following:
Employer: National and Kapodistrian University of Athens; Consultancy: Bayer; Medtronic; Novartis; Servier; Vifor; Boehringer Ingelheim, Impulse Dynamics, Cardior, Merck, Novo Nordisk;; Research Funding: No Pharma or device; European Union; Honoraria: Bayer, Boehringer Ingelheim, Cardior, Merck, Novo Nordisk; Advisory or Leadership Role: JACC HF; EJHF, Past President of Hellenic Heart failure Society; and Speakers Bureau: Boehringer Ingelheim, Bayer, Novo Nordisc.

I understand that the information above will be published within the journal article, if accepted, and that failure to comply and/or to accurately and completely report the potential financial conflicts of interest could lead to the following: 1) Prior to publication, article rejection, or 2) Post-publication, sanctions ranging from, but not limited to, issuing a correction, reporting the inaccurate information to the authors' institution, banning authors from submitting work to ASN journals for varying lengths of time, and/or retraction of the published work.

Name: Gerasimos Filippatos
Manuscript ID: CJASN-2025-000716R1
Manuscript Title: Finerenone in People with Chronic Kidney Disease, Type 2 Diabetes and a History of Nephrectomy: A FIDELITY Analysis
Date of Completion: October 21, 2025
Disclosure Updated Date: October 21, 2025

ASN American Society of Nephrology | JASN Journal of the American Society of Nephrology | KIDNEY360 Accessing Our World From Every Angle | CJASN Clinical Journal of the American Society of Nephrology

ASN Journal Disclosure Form

As per ASN journal policy, I have disclosed any financial relationships or commitments I have held in the past 36 months as included below. I have listed my Current Employer below to indicate there is a relationship requiring disclosure. If no relationship exists, my Current Employer is not listed.

A. Fornoni reports the following:
Employer: University of Miami; Consultancy: Dimerix, Travere, Vera, Alexion, Novartis, Calliditas, Bayer; Ownership Interest: I am CSO and Vice-President of L&F Health LLC.; I am shareholder for Zyversa Therapeutics; I am shareholder of River 3 Renal Corp; I am shareholder of UpToDate; Research Funding: Pfizer; Patents or Royalties: Patent on the use of cyclodextrin for the treatment of kidney diseases.; Patent on the use of small molecule inducers of cholesterol efflux.; I own shares in Zyversa Therapeutics and R3R.; Advisory or Leadership Role: Fortress Biotech, AION; Speakers Bureau: Curio; and Other Interests or Relationships: I am inventor on 5 pending US patents and two published patents.

I understand that the information above will be published within the journal article, if accepted, and that failure to comply and/or to accurately and completely report the potential financial conflicts of interest could lead to the following: 1) Prior to publication, article rejection, or 2) Post-publication, sanctions ranging from, but not limited to, issuing a correction, reporting the inaccurate information to the authors' institution, banning authors from submitting work to ASN journals for varying lengths of time, and/or retraction of the published work.

Name: Alessia Fornoni
Manuscript ID: CJASN-2025-000716R1
Manuscript Title: Finerenone in People with Chronic Kidney Disease, Type 2 Diabetes and a History of Nephrectomy: A FIDELITY Analysis
Date of Completion: October 20, 2025
Disclosure Updated Date: October 20, 2025

ASN
American Society of Nephrology
JASN
Journal of the American Society of Nephrology
KIDNEY360
Accessing Our World From Every Angle
CJASN
Clinical Journal of the American Society of Nephrology

ASN Journal Disclosure Form

As per ASN journal policy, I have disclosed any financial relationships or commitments I have held in the past 36 months as included below. I have listed my Current Employer below to indicate there is a relationship requiring disclosure. If no relationship exists, my Current Employer is not listed.

There is no minimum financial threshold; individuals must disclose all financial relationships, regardless of the amount, with ineligible companies. Individuals must disclose for every category below, regardless of their view of the relevance of the relationship to the activity. ("*Ineligible companies" are those whose primary business is producing, marketing, selling, re-selling, or distributing health care products used by or on patients.*)

Date: 09 Jan 2026

Author Name: Andrea Horvat-Broecker

Manuscript ID:

Manuscript Title: Finerenone in People with Chronic Kidney Disease, Type 2 Diabetes, and History of Nephrectomy

Disclosure Statement: *(including all categories below*)*

Bayer employer

***Categories:** Employer; Ownership Interest; Consultancy; Research Funding; Honoraria; Patents or Royalties; Advisory or Leadership Role; Speakers Bureau; and Other Interests or Relationships

Author Acknowledgment: I understand that the information above will be published within the journal article, if accepted, and that failure to comply and/or to accurately and completely report the potential financial conflicts of interest could lead to the following: 1) Prior to publication, article rejection, or 2) Post-publication, sanctions ranging from, but not limited to, issuing a correction, reporting the inaccurate information to the authors' institution, banning authors from submitting work to ASN journals for varying lengths of time, and/or retraction of the published work.

Author Name and/or Initials: Andrea Horvat-Broecker

ASN American Society of Nephrology · JASN Journal of the American Society of Nephrology · KIDNEY360 Accessing Our World From Every Angle · CJASN Clinical Journal of the American Society of Nephrology

ASN Journal Disclosure Form

As per ASN journal policy, I have disclosed any financial relationships or commitments I have held in the past 36 months as included below. I have listed my Current Employer below to indicate there is a relationship requiring disclosure. If no relationship exists, my Current Employer is not listed.

J. Munoz Mendoza reports the following:
Employer: University of Miami; and Ownership Interest: Apple; Microsoft; Nvidia; Tesla; Amazon.

I understand that the information above will be published within the journal article, if accepted, and that failure to comply and/or to accurately and completely report the potential financial conflicts of interest could lead to the following: 1) Prior to publication, article rejection, or 2) Post-publication, sanctions ranging from, but not limited to, issuing a correction, reporting the inaccurate information to the authors' institution, banning authors from submitting work to ASN journals for varying lengths of time, and/or retraction of the published work.

Name: Jair Munoz Mendoza
Manuscript ID: CJASN-2025-000716R1
Manuscript Title: Finerenone in People with Chronic Kidney Disease, Type 2 Diabetes and a History of Nephrectomy: A FIDELITY Analysis
Date of Completion: August 29, 2025
Disclosure Updated Date: August 29, 2025

ASN
American Society of Nephrology
JASN
Journal of the American Society of Nephrology
KIDNEY360
Accessing Our World From Every Angle
CJASN
Clinical Journal of the American Society of Nephrology

ASN Journal Disclosure Form

As per ASN journal policy, I have disclosed any financial relationships or commitments I have held in the past 36 months as included below. I have listed my Current Employer below to indicate there is a relationship requiring disclosure. If no relationship exists, my Current Employer is not listed.

K. Rohwedder reports the following:
Employer: Bayer AG

I understand that the information above will be published within the journal article, if accepted, and that failure to comply and/or to accurately and completely report the potential financial conflicts of interest could lead to the following: 1) Prior to publication, article rejection, or 2) Post-publication, sanctions ranging from, but not limited to, issuing a correction, reporting the inaccurate information to the authors' institution, banning authors from submitting work to ASN journals for varying lengths of time, and/or retraction of the published work.

Name: Katja Rohwedder
Manuscript ID: CJASN-2025-000716R1
Manuscript Title: Finerenone in People with Chronic Kidney Disease, Type 2 Diabetes and a History of Nephrectomy: A FIDELITY Analysis
Date of Completion: October 30, 2025
Disclosure Updated Date: July 3, 2025

ASN
American Society of Nephrology
JASN
Journal of the American Society of Nephrology
KIDNEY360
Accessing Our World From Every Angle
CJASN
Clinical Journal of the American Society of Nephrology

ASN Journal Disclosure Form

As per ASN journal policy, I have disclosed any financial relationships or commitments I have held in the past 36 months as included below. I have listed my Current Employer below to indicate there is a relationship requiring disclosure. If no relationship exists, my Current Employer is not listed.

P. Rossing reports the following:
Employer: Steno Diabetes Center Copenhagen; Research Funding: Novo Nordisk , AstraZeneca, Bayer, Lexicon Pharma; Honoraria: Boehringer Ingelheim, AstraZeneca, Abbott, Novo Nordisk, all honoraria to institution; and Advisory or Leadership Role: Astra Zeneca Bayer , Novo Nordisk, Gilead all honoraria to institution.

I understand that the information above will be published within the journal article, if accepted, and that failure to comply and/or to accurately and completely report the potential financial conflicts of interest could lead to the following: 1) Prior to publication, article rejection, or 2) Post-publication, sanctions ranging from, but not limited to, issuing a correction, reporting the inaccurate information to the authors' institution, banning authors from submitting work to ASN journals for varying lengths of time, and/or retraction of the published work.

Name: Peter Rossing
Manuscript ID: CJASN-2025-000716R1
Manuscript Title: Finerenone in People with Chronic Kidney Disease, Type 2 Diabetes and a History of Nephrectomy: A FIDELITY Analysis."
Date of Completion: August 29, 2025
Disclosure Updated Date: March 19, 2025

ASN American Society of Nephrology
JASN Journal of the American Society of Nephrology
KIDNEY360 Accessing Our World From Every Angle
CJASN Clinical Journal of the American Society of Nephrology

ASN Journal Disclosure Form

As per ASN journal policy, I have disclosed any financial relationships or commitments I have held in the past 36 months as included below. I have listed my Current Employer below to indicate there is a relationship requiring disclosure. If no relationship exists, my Current Employer is not listed.

M. Weir reports the following:
Employer: University of Maryland School of Medicine; Consultancy: AstraZeneca, Bayer, Novo Nordisk, Vera, Mineralys, CSL Vifor, Medtronic, Intercept, Daxor , Corcept; and Honoraria: Same as above for ad hoc advisory board meetings.

I understand that the information above will be published within the journal article, if accepted, and that failure to comply and/or to accurately and completely report the potential financial conflicts of interest could lead to the following: 1) Prior to publication, article rejection, or 2) Post-publication, sanctions ranging from, but not limited to, issuing a correction, reporting the inaccurate information to the authors' institution, banning authors from submitting work to ASN journals for varying lengths of time, and/or retraction of the published work.

Name: Matthew R. Weir
Manuscript ID: CJASN-2025-000716R1
Manuscript Title: Finerenone in People with CKD, T2DM and a history of nephrectomy: A FIDELIRT Analysis
Date of Completion: August 29, 2025
Disclosure Updated Date: January 6, 2025
