# Supplementary material for: Finerenone in People with CKD, Type 2 Diabetes, and History of Nephrectomy
Source: Clin J Am Soc Nephrol. 2026 Jan 14;21(3):414–24. doi: 10.2215/CJN.0000000932 (PMC12959748; doi:10.2215/CJN.0000000932)
Supplement: Supplementary file 2 [file cjasn-21-414-s002.pdf]

Supplemental Material

Contents

FIDELIO-DKD Investigators 2

FIGARO-DKD Investigators 11

Plain Language Summary 22

Supplemental Tables and Figures 23

Supplemental Table 1. *P* values for the main factors and interaction terms used in the mixed model for urine albumin-to-creatinine ratio to baseline analysis 23

Supplemental Table 2. Analysis of covariance for ratio to baseline of urine albumin-to-creatinine ratio at month 4 (closest visit[a]) 25

Supplemental Table 3. Urine albumin-to-creatinine ratio reductions up to two years according to nephrectomy status in medical history (unadjusted analysis; full analysis set) 27

Supplemental Table 4. Summary of treatment-emergent adverse events by body system or organ class (safety analysis set) 28

Supplemental Figure 1. Time from nephrectomy to randomization. 30

FIDELIO-DKD Investigators

National Lead Investigators

Augusto Vallejos (Argentina), Richard MacIsaac (Australia), Guntram Schernthaner (Austria), Pieter Gillard (Belgium), Maria Eugenia F. Canziani (Brazil), Theodora Temelkova-Kurktschiev (Bulgaria), Ellen Burgess and Sheldon Tobe (Canada), Fernando González (Chile), Zhi-Hong Liu (China), Andrés Ángelo Cadena Bonfanti and Carlos Francisco Jaramillo (Colombia), Martin Prazny (Czech Republic), Peter Rossing (Denmark), Jorma Strand (Finland), Michel Marre (France), Roland Schmieder and Christoph Wanner (Germany), Pantelis A. Sarafidis (Greece), Juliana Chan (Hong Kong), László Rosivall (Hungary), Joseph Eustace (Ireland), Ehud Grossman and Yoram Yagil (Israel), Giuseppe Remuzzi (Italy), Daisuke Koya and Takashi Wada (Japan), Magdalena Madero Rovalo (Mexico), Ron Gansevoort and Adriaan Kooy (Netherlands), Trine Finnes (Norway), Froilan De Leon (Philippines), Janusz Gumprecht (Poland), Fernando Teixeira e Costa (Portugal), Alexander Dreval (Russia), Anantharaman Vathsala (Singapore), Aslam Amod (South Africa), Sin Gon Kim and Byung Wan Lee (South Korea), Julio Pascual Santos (Spain), Bengt-Olov Tengmark (Sweden), Michel Burnier (Switzerland), Chien-Te Lee (Taiwan), Sukit Yamwong (Thailand), Ramazan Sari (Turkey), Kieran McCafferty (United Kingdom), Borys Mankovsky (Ukraine), Sharon Adler, Linda Fried, Robert Toto and Mark Williams (United States), Tran Quang Khanh (Vietnam)

Participating Countries and Investigators

Argentina: Diego Aizenberg, Inés Bartolacci, Diego Besada, Julio Bittar, Mariano Chahin, Alicia Elbert, Elizabeth Gelersztein, Alberto Liberman, Laura Maffei, Federico Pérez Manghi, Hugo Sanabria, Augusto Vallejos, Gloria Viñes, Alfredo Wassermann

Australia: Walter Abhayaratna, Shamasunder Acharya, Elif Ekinci, Darren Lee, Richard MacIsaac, Peak Mann Mah, Craig Nelson, David Packham, Alexia Pape, Simon Roger, Hugo Stephenson, Michael Suranyi, Gary Wittert, Elizabeth Vale

Austria: Martin Clodi, Christoph Ebenbichler, Evelyn Fliesser-Görzer, Ursula Hanusch, Michael Krebs, Karl Lhotta, Bernhard Ludvik, Gert Mayer, Peter Neudorfer, Bernhard Paulweber, Rudolf Prager, Wolfgang Preiß, Friedrich Prischl, Gerit-Holger Schernthaner, Harald Sourij, Martin Wiesholzer

Belgium: Peter Doubel, Wendy Engelen, Pieter Gillard, Jean-Michel Hougardy, Jean-Marie Krzesinski, Bart Maes, Marijn Speeckaert, Koen Stas, Luc van Gaal, Hilde Vanbelleghem

Brazil: Daniela Antunes, Roberto Botelho, Claudia Brito, Luis Canani, Maria Eugenia Canziani, Maria Cerqueira, Rogerio de Paula, Freddy Eliaschewitz, Carlos Eduardo Figueiredo, Adriana Forti, Miguel Hissa, Maurilo Leite Jr, Emerson Lima, Irene Noronha, Bruno Paolino, Nathalia Paschoalin, Raphael Paschoalin, Roberto Pecoits Filho, Marcio Pereira, Evandro Portes, Dalton Precoma, Rosangela Rea, Miguel Riella, Joao Eduardo Salles, Eduardo Vasconcellos, Sergio Vencio

Bulgaria: Emiliya Apostolova, Radostina Boshnyashka, Ghassan Farah, Dimitar Georgiev, Valentina Gushterova, Neli Klyuchkova, Mariya Lucheva, Petya Manova, Dotska Minkova, Boyan Nonchev, Mariyana Pichmanova, Zhulieta Prakova, Rangel Rangelov, Rosen Rashkov, Pavel Stanchev, Bilyana Stoyanovska-Elencheva, Zhivko Tagarev, Theodora Temelkova-Kurktschieva, Svetla Vasileva, Mariana Yoncheva-Mihaylova

Canada: Paul Barre, Brian Carlson, James Conway, Serge Cournoyer, Richard Dumas, Sameh Fikry, Richard Goluch, Pavel Hamet, Randolph Hart, Sam Henein, Joanne Liutkus, Francois Madore, Valdemar Martinho, Giuseppe Mazza, Philip McFarlane, Dennis O' Keefe, Sean Peterson, Daniel Schwartz, Daniel Shu, Andrew Steele, Guy Tellier, Karthik Tennankore, Sheldon Tobe, George Tsoukas, Richard Tytus, Louise Vitou, Michael Walsh, Stanley Weisnagel, Igor Wilderman, Jean-Francois Yale

Chile: Jorge Cobos, Juan Godoy, Fernando González, Sergio Lobos, Juan Carlos Palma, Juan Carlos Prieto, Eliana Reyes, Carmen Romero, Victor Saavedra, Mario Vega

China: Ruifang Bu, Hanqing Cai, Nan Chen, Qinkai Chen, Dejun Chen, Jinluo Cheng, Youping Dong, Junwu Dong, Tianjun Guan, Chuanming Hao, Wen Huang, Fangfang Jiang, Minxiang Lei, Ling Li, Zhonghe Li, Xuemei Li, Jingmei Li, Yan Li, Xinling Liang, Bo Liang,

Fang Liu, Yinghong Liu, Yuantao Liu, Zhihong Liu, Gang Long, Guoyuan Lu, Weiping Lu, Yibing Lu, Ping Luo, Jianhua Ma, Zhaohui Mo, Jianying Niu, Ai Peng, Jiansong Shen, Feixia Shen, Bingyin Shi, Qing Su, Zhuxing Sun, Shuifu Tang, Nanwei Tong, Hao Wang, Xinjun Wang, Lihua Wang, Guixia Wang, Jianqin Wang, Yangang Wang, Li Wang, Jiali Wei, Tianfeng Wu, Chaoqing Wu, Changying Xing, Fei Xiong, Xudong Xu, Ning Xu, Tiekun Yan, Jinkui Yang, Aiping Yin, Longyi Zeng, Hao Zhang, Yanlin Zhang, Ying Zhang, Wenjing Zhao, Zhiquan Zhao, Hongguang Zheng, Ling Zhong, Dalong Zhu, Yongze Zhuang

Colombia: Clara Arango, Sandra Barrera, Nelly Beltrán López, Diego Benitez, Guillermo Blanco, Andrés Cadena, Julian Coronel, Carlos Cure, Carlos Durán, Alexander González, Gustavo Guzmán, Eric Hernández, Jaime Ibarra, Carlos Jaramillo, Nicolás Jaramillo, William Kattah, Dora Molina, Gregorio Sánchez, Mónica Terront, Freddy Trujillo, Miguel Urina, Ruben Vargas, Iván Villegas, Hernán Yupanqui

Czech Republic: Dino Alferi, Michal Brada, Jiri Brezina, Petr Bucek, Tomas Edelsberger, Drahomira Gulakova, Jitka Hasalova Zapletalova, Olga Hola, Lucie Hornova, Jana Houdova, Helena Hrmova, David Karasek, Sarka Kopecka, Richard Kovar, Eva Krcova, Jiri Kuchar, Vlasta Kutejova, Hana Lubanda, Ivo Matyasek, Magdalena Mokrejsova, Libor Okenka, Martin Prazny, Jiri Pumprla, Pavel Tomanek

Denmark: Jesper Bech, Jens Faber, Gunnar Gislason, Jørgen Hangaard, Grzegorz Jaroslaw Pacyk, Claus Juhl, Thure Krarup, Morten Lindhardt, Sten Madsbad, Joan Nielsen, Ulrik Pedersen-Bjergaard, Per Poulsen, Ole Rasmussen, Peter Rossing, Karoline Schousboe

Finland: Mikko Honkasalo, Mikko Honkasalo, Kari Humaloja, Kristiina Kananen, Ilkka Kantola, Arvo Koistinen, Pirkko Korsoff, Jorma Lahtela, Sakari Nieminen, Tuomo Nieminen, Karita Sadeharju, Jorma Strand, Sakari Sulosaari

France: Bertrand Cariou, François Chantrel, Sylvaine Clavel, Christian Combe, Jean-Pierre Fauvel, Karim Gallouj, Didier Gouet, Bruno Guerci, Dominique Guerrot, Maryvonne Hourmant, Alexandre Klein, Christophe Mariat, Michel Marre, Rafik Mesbah, Yannick Le

Meur, Arnaud Monier, Olivier Moranne, Pierre Serusclat, Benoit Vendrely, Bruno Verges, Philippe Zaoui

Germany: Christoph Axthelm, Andreas Bergmann, Andreas L. Birkenfeld, Hermann Braun, Klaus Busch, Christel Contzen, Stefan Degenhardt, Karl Derwahl, Thomas Giebel, Andreas Hagenow, Hermann Haller, Christoph Hasslacher, Thomas Horacek, Wolfgang Jungmair, Christof Kloos, Thorsten Koch, Thilo Krüger, Anja Mühlfeld, Joachim Müller, Andreas Pfützner, Frank Pistrosch, Ludger Rose, Lars Rump, Volker Schettler, Ingolf Schiefke, Heike Schlichthaar, Bernd Schröppel, Thomas Schürholz, Helena Sigal, Lutz Stemler, Georg Strack, Heidrun Täschner, Nicole Toursarkissian, Diethelm Tschöpe, Achim Ulmer, Markus van der Giet, Christoph Wanner, Bernhard R. Winkelmann

Greece: Ioannis Boletis, Erifili Hatziagelaki, Ioannis Ioannidis, Theodora Kounadi, Ioanna Makriniotou, Dorothea Papadopoulou, Aikaterini Papagianni, Ploumis Passadakis, Ioannis Stefanidis

Hong Kong: Tai Pang Ip, Paul Lee, On Yan Andrea Luk, Angela Wang, Vincent Yeung

Hungary: Dora Bajcsi, Peter Danos, Eleonora Harcsa, Szilvia Kazup, Katalin Keltai, Robert Kirschner, Julianna Kiss, Laszlo Kovacs, Beata Lamboy, Botond Literati-Nagy, Margit Mileder, Laszlo Nagy, Ebrahim Noori, Gabor Nyirati, Gizella Petro, Karoly Schneider, Albert Szocs, Szilard Vasas, Krisztina Wudi, Zsolt Zilahi, Marianna Zsom

Ireland: Joe Eustace, John Holian, Donal Reddan, Yvonne O' Meara

Israel: Rosane Abramof Ness, Faiad Adawi, Zaher Armaly, Shaul Atar, Sydney Ben Chetrit, Noa Berar Yanay, Gil Chernin, Mahmud Darawsha, Shai Efrati, Mazen Elias, Evgeny Farber, Mariela Glandt, Ehud Grossman, Majdi Halabi, Khaled Khazim, Idit Liberty, Ofri Mosenzon, Assy Nimer, Doron Schwartz, Julio Wainstein, Yoram Yagil, Robert Zukermann

Italy: Angelo Avogaro, Giovanni Giorgio Battaglia, Enzo Bonora, Carlo Antonio Bossi, Paolo Calabrò, Franco Luigi Cavalot, Roberto Cimino, Mario Gennaro Cozzolino, Enrico Fiaccadori, Paolo Fiorina, Carlo Bruno Giorda, Maria Cristina Gregorini, Gaetano La Manna, Davide Carlo Maggi, Antonello Pani, Norberto Perico, PierMarco Piatti, Antonio Pisani, Paola

Ponzani, Gennaro Santorelli, Domenico Santoro, Giancarlo Tonolo, Roberto Trevisan, Anna Maria Veronelli

Japan: Hideo Araki, Osamu Ebisui, Naruhiro Fujita, Ryuichi Furuya, Yoshiyuki Hamamoto, Masahiro Hatazaki, Terumasa Hayashi, Takayuki Higashi, Yoshihide Hirohata, Shuji Horinouchi, Masayuki Inagaki, Masao Ishii, Tamayo Ishiko, Hideaki Jinnouchi, Hidetoshi Kanai, Daisuke Kanda, Hideo Kanehara, Masayuki Kashima, Kiyoe Kato, Takeshi Katsuki, Katsunori Kawamitsu, Satsuki Kawasaki, Fumi Kikuchi, Hidetoshi Kikuchi, Kunihisa Kobayashi, Junko Koide, Miyuki Kubota, Yoshiro Kusano, Hajime Maeda, Sunao Matsubayashi, Kazunari Matsumoto, Yasuto Matsuo, Naoki Matsuoka, Hiroaki Miyaoka, Satoshi Murao, Mikihiro Nakayama, Jun Nakazawa, Takashi Nomiyama, Masayuki Noritake, Takayuki Ogiwara, Hiroshi Ohashi, Hideki Okamoto, Takeshi Osonoi, Nobuhiro Sasaki, Taiji Sekigami, Taro Shibasaki, Hirotaka Shibata, Junji Shinoda, Hiroshi Sobajima, Kazuya Sugitatsu, Toshiyuki Sugiura, Toru Sugiyama, Daisuke Suzuki, Hiroyuki Suzuki, Masaaki Suzuki, Asami Takeda, Asami Tanaka, Seiichi Tanaka, Izumi Tsunematsu, Makoto Ujihara, Daishiro Yamada, Masayo Yamada, Kazuo Yamagata, Ken Yamakawa, Fumiko Yamakawa, Yoshimitsu Yamasaki, Yuko Yambe, Taihei Yanagida, Hidekatsu Yanai, Tetsuyuki Yasuda

Lithuania: Dovile Kriauciuniene, Jurate Lasiene, Antanas Navickas, Lina Radzeviciene, Egle Urbanaviciene, Gediminas Urbonas, Audrone Velaviciene

Malaysia: Rohana Abd Ghani, Nor Azizah Aziz, Li Yuan Lee, Chek Loong Loh, Norhaliza Mohd Ali, Nurain Mohd Noor, Nik Nur Fatnoon Nik Ahmad, Jeyakantha Ratnasingam, Wan Hasnul Halimi Bin Wan Hasan, Wan Mohd Izani Wan Mohamed

Mexico: Sandro Avila Pardo, Miriam Bastidas Adrian, Alfredo Chew Wong, Jorge Escobedo de la Peña, Guillermo Fanghänel Salmón, Guillermo González Gálvez, Ramiro Gutiérrez Ochoa, Saúl Irizar Santana, Magdalena Madero Rovalo, Gustavo Méndez Machado, Luis Nevarez Ruiz, Denisse Ramos Ibarra, Gabriel Ramos López, Leobardo Sauque Reyna, Gustavo Solache Ortiz, Rafael Valdez Ortiz, Juan Villagordoa Mesa

Netherlands: R.C. Bakker, J.N.M. Barendregt, A.H. Boonstra, Willem Bos, C.B. Brouwer, M. van Buren, Ron Gansevoort, Adriaan Kooy, Marielle Krekels, Ruud J.M. van Leendert, Louis A.G. Lieverse, P.T. Luik, E. Lars Penne, Peter Smak Gregoor, Liffert Vogt

New Zealand: John Baker, Veronica Crawford, Rick Cutfield, Peter Dunn, Jeremy Krebs, Kingsley Nirmalaraj, Russell Scott, Nine Smuts

Norway: Erik Eriksen, Trine Finnes, Hans Høivik, Thomas Karlsson, Peter Scott Munk, Maria Radtke, Knut Risberg, Jan Rocke, Leidulv Solnør, Aud-Eldrid Stenehjem, Anne-Beathe Tafjord

Philippines: Glenda Pamugas, Araceli Panelo, Ronald Perez, Maribel Tanque, Louie Tirador, Michael Villa

Poland: Patrycja Butrymowicz, Kazimierz Ciechanowski, Grazyna Cieslik, Edward Franek, Janusz Gumprecht, Michal Hoffmann, Jolanta Krzykowska, Ilona Kurnatowska, Katarzyna Landa, Adam Madrzejewski, Katarzyna Madziarska, Stanislaw Mazur, Piotr Napora, Michal Nowicki, Anna Ocicka-Kozakiewicz, Barbara Rewerska, Teresa Rusicka, Jan Ruxer, Ewa Skokowska, Andrzej Stankiewicz, Tomasz Stompor, Agnieszka Tiuryn-Petrulewicz, Katarzyna Wasilewska, Bogna Wierusz-Wysocka, Renata Wnetrzak-Michalska

Portugal: Edgar Almeida, Rosa Ballesteros, Carlos Barreto, Idalina Beirao, Rita Birne, Cesar Esteves, Jose Guia, Susana Heitor, Olinda Marques, Pedro Melo, Fernando Nolasco, Amalia Pereira, Cristina Roque, Francisco Rosario, Gil Silva, Ana Silva, Fernando Teixeira e Costa, Ana Vila Lobos

Puerto Rico: Gregorio Cortes-Maisonet, Amaury Roman-Miranda

Romania: Adrian Albota, Cornelia Bala, Hortensia Barbonta, Elena Caceaune, Doina Catrinoui, Ciprian Constantin, Adriana Dumitrescu, Nicoleta Mindrescu, Cristina Mistode, Gabriela Negrisanu, Adriana Onaca, Silvia Paveliu, Ella Pintilei, Lavinia Pop, Amorin Popa, Alexandrina Popescu, Gabriela Radulian, Iosif Szilagyi, Liana Turcu, Georgeta Vacaru, Adrian Vlad

Russia: Mikhail Antsiferov, Mikhail Arkhipov, Andrey Babkin, Olga Barbarash, Vitaliy Baranov, Elena Chernyavskaya, Arkadiy Demko, Alexander Dreval, Anton Edin, Polina

Ermakova, Valentin Fadeev, Albert Galyavich, Leyla Gaysina, Ivan Gordeev, Irina Ipatko, Marina Kalashnikova, Yuriy Khalimov, Vadim Klimontov, Zhanna Kobalava, Elena Kosmacheva, Natalya Koziolova, Lyudmila Kvitkova Sergey Levashov, Roman Libis, Vyacheslav Marasaev, Natalia Malykh, Vladimir Martynenko, Sofya Malyutina, Imad Merai, Ashot Mkrtumyan, Galina Nechaeva, Nina Petunina, Shamil Palyutin, Leonid Pimenov, Elena Rechkova, Tatyana Rodionova, Oksana Rymar, Ruslan Sardinov, Olga Semenova, Alexander Sherenkov, Oleg Solovev, Elena Smolyarchuk, Leonid Strongin, Olga Ukhanova, Nadezhda Verlan, Natalya Vorokhobina, Davyd Yakhontov, Sergey Yakushin, Elena Zakharova, Alsu Zalevskaya, Olga Zanozina, Elena Zhdanova, Larisa Zhukova, Tatyana Zykova

Singapore: Chee Fang Sum, Sufi Muhummad Suhail, Ru San Tan, Anantharaman Vathsala, Edmund Wong

Slovakia: Jana Babikova, Ingrid Buganova, Andrej Dzupina, Zuzana Ochodnicka, Dalibor Sosovec, Denisa Spodniakova

South Africa: Fayzal Ahmed, Aslam Amod, Sindeep Bhana, Larry Distiller, Dirkie Jansen van Rensburg, Mukesh Joshi, Shaifali Joshi, Deepak Lakha, Essack Mitha, Gracjan Podgorski, Naresh Ranjith, Brian Rayner, Paul Rheeder, Mohamed Sarvan, Mary Seeber, Heidi Siebert, Mohammed Tayob, Julien Trokis, Dorothea Urbach, Louis van Zyl

South Korea: Bum-Soon Choi, Moon Gi Choi, ChoonHee Chung, YouCheol Hwang, ChongHwa Kim, InJoo Kim, JaeHyeon Kim, SinGon Kim, SungGyun Kim, Tae Hee Kim, WooJe Lee, ByungWan Lee, Kang Wook Lee, Kook-Hwan Oh, Ji Eun Oh, Yun Kyu Oh, Dong-Jin Oh, Junbeom Park, Seok Joon Shin, Su-Ah Sung, Jae Myung Yu

Spain: Irene Agraz, Francisco Javier Ampudia, Hanane Bouarich, Francesca Calero, Cristina Castro, Secundino Cigarrán Guldris, Josep Cruzado Garrit, Fernando de Álvaro, Josep Galcerán, Olga González Albarrán, Julio Hernández Jaras, Meritxell Ibernón, Francisco Martínez Deben, Mª Dolores Martínez Esteban, José María Pascual Izuel, Judith Martins, Juan Mediavilla, Alfredo Michán, Julio Pascual Santos, Esteban Poch, Manuel

Polaina Rusillo, Carlos Sánchez Juan, Rafael Santamaría Olmo, José Julián Segura de la Morena, Alfonso Soto, Maribel Troya

Sweden: Annette Bruchfeld, Dan Curiac, Ken Eliasson, Malin Frank, Gregor Guron, Olof Hellberg, Margareta Hellgren, Hans Larnefeldt, Carl-Johan Lindholm, Magnus Löndahl, Erik Rein-Hedin, Inga Soveri, Jonas Spaak, Bengt-Olov Tengmark

Switzerland: Daniel Ackermann, Stefan Bilz, Michel Burnier, Christian Forster, Stefan Kalbermatter, Andreas Kistler, Antoinette Pechère-Bertschi, Bernd Schultes

Taiwan: Chiz-Tzung Chang, Cheng-Chieh Hung, Ju-Ying Jiang, Chien-Te Lee, Shuei-Liong Lin, Der-Cherng Tarng, Shih-Te Tu, Mai-Szu Wu, Ming-Ju Wu

Thailand: Chaicharn Deerochanawong, Chagriya Kitiyakara, Vuddhidej Ophascharoensuk, Chatlert Pongchaiyakul, Bancha Satirapoj

Turkey: Necmi Eren, Ibrahim Gul, Okan Gulel, Ismail Kocyigit, Abdulbaki Kumbasar, Idris Sahin, Ramazan Sari, Burak Sayin, Talat Tavli, Sedat Ustundag, Yavuz Yenicerioglu

Ukraine: Iryna Bondarets, Volodymyr Botsyurko, Viktoriia Chernikova, Oleksandra Donets, Ivan Fushtey, Mariia Grachova, Anna Isayeva, Dmytro Kogut, Julia Komisarenko, Nonna Kravchun, Kateryna Malyar, Borys Mankovsky, Liliya Martynyuk, Vitaliy Maslyanko, Halyna Myshanych, Larysa Pererva, Nataliia Pertseva, Oleksandr Serhiyenko, Ivan Smirnov, Liubov Sokolova, Vasyl Stryzhak, Maryna Vlasenko

United Kingdom: Ahmad AbouSaleh, Jonathan Barratt, Cuong Dang, Hassan Kahal, Adam Kirk, Anne Kilvert, Sui Phi Kon, Kieran McCafferty, Dipesh Patel, Sam Rice, Arutchelvam Vijayaraman, Yuk-ki Wong, Martin Gibson, Mona Wahba, Reza Zaidi

United States: Idalia Acosta, Atoya Adams, Sharon Adler, Dilawar Ajani, Slamat Ali, Radica Alicic, Amer Al-Karadsheh, Sreedhara Alla, D. Allison, Nabil Andrawis, Ahmed Arif, Ahmed Awad, Masoud Azizad, Michael Bahrami, Shweta Bansal, Steven Barag, Ahmad Barakzoy, Mark Barney, Joshua Barzilay, Khalid Bashir, Jose Bautista, Srinivasan Beddhu, Diogo Belo, Sabrina Benjamin, Ramin Berenji, Anuj Bhargava, Jose Birriel, Stephen Brietzke, Frank Brosius, Osvaldo Brusco, Anna Burgner, Robert Busch, Rafael Canadas, Maria Caramori, Jose Cardona, Christopher Case, Humberto Cruz, Ramprasad Dandillaya, Dalia Dawoud,

Zia Din, Bradley Dixon, Ankur Doshi, James Drakakis, Mahfouz El Shahawy, Ashraf El-Meanawy, Mohammed El-Shahawy, John Evans, George Fadda, Umar Farooq, Roland Fernando, Raymond Fink, Brian First, David Fitz-Patrick, John Flack, Patrick Fluck, Leon Fogelfeld, Vivian Fonseca, Juan Frias, Claude Galphin, Luis Garcia-Mayol, Gary Goldstein, Edgar Gonzalez, Francisco Gonzalez-Abreu, Ashwini Gore, David Grant, Violet Habwe, Maxine Hamilton, Jamal Hammoud, Stuart Handelsman, Israel Hartman, Glenn Heigerick, Andrew Henry, German Hernandez, Carlos Hernandez-Cassis, Carlos Herrera, Joachim Hertel, Wenyu Huang, Rogelio Iglesias, Ali Iranmanesh, Timothy Jackson, Mahendra Jain, Kenneth Jamerson, Karen Johnson, Eric Judd, Joshua Kaplan, Zeid Kayali, Bobby Khan, Muhammad Khan, Sourabh Kharait, M. Sue Kirkman, Nelson Kopyt, Wayne Kotzker, Csaba Kovesdy, Camil Kreit, Arvind Krishna, Saeed Kronfli, Keung Lee, Derek LeJeune, Brenda Lemus, Carlos Leon-Forero, Douglas Linfert, Henry Lora, Alexander Lurie, Geetha Maddukuri, Alexander Magno, Louis Maletz, Sreedhar Mandayam, Mariana Markell, Ronald Mayfield, Caroline Mbogua, Dierdre McMullen, Carl Meisner, Stephen Minton, Bharat Mocherla, Rajesh Mohandas, Manuel Montero, Moustafa Moustafa, Salil Nadkarni, Samer Nakhle, Jesus Navarro, Nilda Neyra, Romanita Nica, Philip Nicol, Paul Norwood, Visal Numrungroad, Richard O' Donovan, A. Odugbesan, Jorge Paoli-Bruno, Samir Parikh, Rakesh Patel, Aldo Peixoto, Pablo Pergola, Alan Perlman, Karlton Pettis, Roberto Pisoni, Mirela Ponduchi, Jorge Posada, Sharma Prabhakar, Jai Radhakrishnan, Mahboob Rahman, Rupesh Raina, Anjay Rastogi, Efrain Reisin, Marc Rendell, David Robertson, Michael Rocco, Hugo Romeu, Sylvia Rosas, Jack Rosenfeld, Dennis Ross, Jeffrey Rothman, Lance Rudolph, Yusuf Ruhullah, Gary Ruoff, Jeffrey Ryu, Mandeep Sahani, Ramin Sam, Garfield Samuels, William Sanchez, Vladimir Santos, Scott Satko, Sanjeev Saxena, David Scott, Gilberto Seco, Melvin Seek, Harvey Serota, Tariq Shafi, Nauman Shahid, Michael Shanik, Santosh Sharma, Arjun Sinha, James Smelser, Mark Smith, Kyaw Soe, Richard Solomon, Eugene Soroka, Joseph Soufer, Bruce Spinowitz, Leslie Spry, Rosa Suarez, Bala Subramanian, Harold Szerlip, Aparna Tamirisa, Stephen Thomson, Tuan-Huy Tran, Richard Treger, Gretel Trullenque, Thomas Turk, Guillermo Umpierrez, Daniel Urbach, Martin

Valdes, Shujauddin Valika, Damaris Vega, Peter Weissman, Adam Whaley-Connell, Jonathan Winston, Jonathan Wise, Alan Wynne, Steven Zeig

Vietnam: Phuong Chu, Lam Van Hoang, Tran Khanh, Nguyen Thi Phi Nga, Pham Nguyen Son, Lan Phuong Tran

FIGARO-DKD Investigators

Steering Committee Members

Augusto Vallejos (Argentina); Richard MacIsaac (Australia); Guntram Schernthaner (Austria); Pieter Gillard (Belgium); Maria Eugenia F. Canziani (Brazil);
Theodora Temelkova-Kurktschiev (Bulgaria); Ellen Burgess and Sheldon Tobe (Canada); Fernando González (Chile); Zhi-Hong Liu (China); Andrés Ángelo Cadena Bonfanti and Carlos Francisco Jaramillo (Colombia); Martin Prazny (Czech Republic); Peter Rossing (Denmark); Jorma Strand (Finland); Michel Marre (France); Roland Schmieder and Christoph Wanner (Germany); Pantelis A. Sarafidis (Greece); Juliana Chan (Hong Kong); László Rosivall (Hungary); Joseph Eustace (Ireland); Ehud Grossman and Yoram Yagil (Israel); Giuseppe Remuzzi (Italy); Daisuke Koya and Takashi Wada (Japan);
Luis Alejandro Nevarez Ruiz (Mexico); Ron Gansevoort and Adriaan Kooy (Netherlands); Trine Finnes (Norway); Froilan De Leon (Philippines); Janusz Gumprecht (Poland); Fernando Teixeira e Costa (Portugal); Alexander Dreval (Russia); Anantharaman Vathsala (Singapore); Aslam Amod (South Africa); Sin Gon Kim and Byung Wan Lee (South Korea); Julio Pascual Santos (Spain); Bengt-Olov Tengmark (Sweden); Michel Burnier (Switzerland); Chien-Te Lee (Taiwan); Sukit Yamwong (Thailand); Ramazan Sari (Turkey);
Kieran McCafferty (United Kingdom); Borys Mankovsky (Ukraine); Sharon Adler, Linda Fried, Robert Toto, and Mark Williams (United States); Tran Quang Khanh (Vietnam).

Members: Rajiv Agarwal (Chair), Stefan Anker, Phyllis August, Andrew Coats, Hans Diener, Wolfram Döhner, Barry Greenberg, Stephan von Haehling, James Januzzi, Alan Jardine,

Carlos Kase, Sankar Navaneethan, Lauren Phillips, Piotr Ponikowski, Pantelis Sarafidis, Titte Srinivas, Turgut Tatlisumak, and John Teerlink.

Participating Countries and Investigators

Argentina: Diego Aizenberg, Inés Bartolacci, Diego Besada, Julio Bittar, Mariano Chahin, Alicia Elbert, Elizabeth Gelersztein, Alberto Liberman, Laura Maffei, Federico Pérez Manghi, Hugo Sanabria, Augusto Vallejos, Gloria Viñes, and Alfredo Wassermann.

Australia: Walter Abhayaratna, Peter Colman, David Colquhoun, Elif Ekinci, Chris Ellis, Kim Joshua, Richard MacIsaac, Peak Mann, Craig Nelson, David Packham, Alexia Pape, Eugenia Pedagogos, Paul Regal, Simon Roger, Hugo Stephenson, Michael Suranyi, Duncan Topliss, James Vandeleur, Johan Verjans, Gary Wittert, and Katie-Jane Wynne.

Austria: Martin Clodi, Heinz Drexel, Christoph Ebenbichler, Evelyn Fliesser-Görzer, Ursula Hanusch, Bernhard Ludvik, Gert Mayer, Peter Neudorfer, Rainer Oberbauer, Bernhard Paulweber, Rudolf Prager, Friedrich Prischl, Gerit-Holger Schernthaner, Hans-Robert Schönherr, and Harald Sourij.

Belgium: Peter Doubel, Francis Duyck, Pieter Gillard, Jean-Michel Hougardy, André Scheen, Marijn Speeckaert, Luc van Gaal, and Hilde Vanbelleghem.

Brazil: Daniela Antunes, Marcelo Bacci, Roberto Botelho, Claudia Brito, Luis Canani, Maria Eugenia Canziani, Maria Cerqueira, Rogerio de Paula, Freddy Eliaschewitz, Carlos Eduardo Figueiredo, Adriana Forti, Miguel Hissa, Maurilo Leite Jr, Lilia Maia, Irene Noronha, Bruno Paolino, Roberto Pecoits Filho, Marcio Pereira, Evandro Portes, Dalton Precoma, Rosangela Rea, Miguel Riella, Joao Eduardo Salles, Eduardo Vasconcellos, Sergio Vencio, and Aline Villacorta.

Bulgaria: Radostina Boshnyashka, Ghassan Farah, Dimitar Georgiev, Valentina Gushterova, Neli Klyuchkova, Mariya Lucheva, Petya Manova, Angel Marinchev, Dotska Minkova, Mariya Miteva, Boyan Nonchev, Mariyana Pichmanova, Zhulieta Prakova, Rangel Rangelov, Rosen Rashkov, Pavel Stanchev, Bilyana Stoyanovska-Elencheva,

Zhivko Tagarev, Theodora Temelkova-Kurktschieva, Svetla Vasileva, and Mariana Yoncheva-Mihaylova.

Canada: Brian Carlson, James Conway, Serge Cournoyer, Richard Dumas, Fadia El Boreky, Sameh Fikry, Richard Goluch, Pavel Hamet, Randolph Hart, Sam Henein, Alan Kelly, Lawrence Leiter, Joanne Liutkus, Francois Madore, Valdemar Martinho, Giuseppe Mazza, Philip McFarlane, Dennis O'Keefe, Sean Peterson, Daniel Schwartz, Daniel Shu, Andrew Steele, Ivor Teitelbaum, Guy Tellier, Karthik Tennankore, Sheldon Tobe, George Tsoukas, Richard Tytus, Louise Vitou, Michael Walsh, Stanley Weisnagel, Igor Wilderman, and Jean-Francois Yale.

Chile: Jorge Cobos, Fernando González, Marcelo Medina, Juan Carlos Prieto Dominguez, Eliana Reyes, Carmen Romero, Victor Saavedra, and Paola Varleta.

China: Ruifang Bu, Hanqing Cai, Nan Chen, Qinkai Chen, Dejun Chen, Jinluo Cheng, Junwu Dong, Youping Dong, Yuming Du, Yi Fang, Tianjun Guan, Weiying Guo, Chuanming Hao, Fangfang Jiang, Sheng Jiang, Jian Kuang, Minxiang Lei, Dongmei Li, Hongmei Li, Jingmei Li, Ling Li, Yan Li, Yinan Li, Yuxiu Li, Zhonghe Li, Fang Liu, Jian Liu, Yinghong Liu, Yu Liu, Zhihong Liu, Yuantao Liu, Gang Long, Guoyuan Lu, Weiping Lu, Yibing Lu, Jianhua Ma, Heng Miao, Zhaohui Mo, Jianying Niu, Ai Peng, Wen Peng, Feixia Shen, Jiansong Shen, Bingyin Shi, Qing Su, Zhuxing Sun, Shuifu Tang, Nanwei Tong, Guixia Wang, Hao Wang, Jianqin Wang, Li Wang, Lihua Wang, Xinjun Wang, Jiali Wei, Chaoqing Wu, Tianfeng Wu, Changying Xing, Fei Xiong, Mingtong Xu, Ning Xu, Xudong Xu, Jinkui Yang, Aiping Yin, Longyi Zeng, Hao Zhang, Yanlin Zhang, Ying Zhang, Zhiquan Zhao, Hongguang Zheng, Ling Zhong, Liyong Zhong, Dalong Zhu, and Jun Zhu.

Colombia: Clara Arango, Edgar Arcos, Gustavo Aroca, Sandra Barrera, Germán Barreto, Nelly Beltrán López, Diego Benitez, Andres Bermudez, Guillermo Blanco, Rodrigo Botero, Tatiana Cárdenas, Julian Coronel, Carlos Cure, Carlos Durán, Wilmer Figueroa, Luis García, Gustavo Guzmán, Eric Hernández, Jaime Ibarra, Carlos Jaramillo, Mónica Jaramillo, Nicolás Jaramillo, William Kattah, Manuel Liévano, Mónica López, Dora Molina,

Ricardo Rosero, Gregorio Sánchez, Mónica Terront, Pedro Trillos, Freddy Trujillo, Miguel Urina, Iván Villegas, and Hernán Yupanqui.

Czech Republic: Dino Alferi, Michal Brada, Petr Bucek, Tomas Edelsberger, Drahomira Gulakova, Jitka Hasalova Zapletalova, Olga Hola, Lucie Hornova, Jana Houdova, Helena Hrmova, David Karasek, Sarka Kopecka, Richard Kovar, Eva Krcova, Jiri Kuchar, Vlasta Kutejova, Hana Lubanda, Ivo Matyasek, Magdalena Mokrejsova, Libor Okenka, Martin Prazny, Jiri Pumprla, and Pavel Tomanek.

Denmark: Ulla Andersen, Alin Andries, Jesper Bech, Jens Faber, Gunnar Gislason, Jeppe Gram, Jørgen Hangaard, Claus Juhl, Thure Krarup, Thomas Lauridsen, Morten Lindhardt, Sten Madsbad, Joan Nielsen, Torben Østergaard, Grzegorz Pacyk, Erling Pedersen, Ulrik Pedersen-Bjergaard, Per Poulsen, Ole Rasmussen, Peter Rossing, Karoline Schousboe, and Birger Thorsteinsson.

Finland: Päivi Flöjt, Mikko Honkasalo, Kari Humaloja, Kristiina Kananen, Ilkka Kantola, Arvo Koistinen, Pirkko Korsoff, Jorma Lahtela, Sakari Nieminen, Karita Sadeharju, Jorma Strand, and Sakari Sulosaari.

France: Bertrand Cariou, François Chantrel, Sylvaine Clavel, Jean-Pierre Fauvel, Karim Gallouj, Bruno Guerci, Dominique Guerrot, Alexandre Klein, Yannick Le Meur, Michel Marre, Rafik Mesbah, Arnaud Monier, Olivier Moranne, Ronan Roussel, Pierre Serusclat, Bruno Verges, and Philippe Zaoui.

Germany: Christoph Axthelm, Andreas Bergmann, Andreas L. Birkenfeld, Hermann Braun, Klaus Busch, Christel Contzen, Stefan Degenhardt, Karl Derwahl, Thomas Giebel, Andreas Hagenow, Hermann Haller, Christoph Hasslacher, Thomas Horacek, Wolfgang Jungmair, Christof Kloos, Thorsten Koch, Annemone Koechel, Thilo Krüger, Joachim Müller, Andreas Pfützner, Andrea Rinke, Ludger Rose, Lars Rump, Volker Schettler, Ingolf Schiefke, Heike Schlichthaar, Norbert Schöll, Kristin Schubert, Thomas Schürholz, Helena Sigal, Lutz Stemler, Georg Strack, Heidrun Täschner, Nicole Toursarkissian, Diethelm Tschöpe, Achim Ulmer, Markus van der Giet, Christoph Wanner, and Bernhard R. Winkelmann.

Greece: Ioannis Boletis, George Dimitriadis, Erifili Hatziagelaki, Christos Iatrou, Ioannis Ioannidis, Theodora Kounadi, Ioanna Makriniotou, Dorothea Papadopoulou, Aikaterini Papagianni, Ploumis Passadakis, George Piaditis, and Ioannis Stefanidis.

Hong Kong: Paul Lee, Ronald Ma, Tai Pang Ip, Wing Sun Chow, and Vincent Yeung.

Hungary: Dora Bajcsi, Peter Danos, Eleonora Harcsa, Akos Kalina, Szilvia Kazup, Katalin Keltai, Robert Kirschner, Julianna Kiss, Laszlo Kovacs, Beata Lamboy, Botond Literati-Nagy, Laszlo Nagy, Ebrahim Noori, Gabor Nyirati, Gizella Petro, Karoly Schneider, Judit Simon, Albert Szocs, Szilard Vasas, Krisztina Wudi, Zsolt Zilahi, and Marianna Zsom.

Ireland: Joe Eustace, John Holian, and Donal Reddan.

Israel: Rosane Abramof Ness, Faiad Adawi, Zaher Armaly, Shaul Atar, Amir Bashkin, Sydney Ben Chetrit, Gil Chernin, Mahmud Darawsha, Shai Efrati, Mazen Elias, Evgeny Farber, Mariela Glandt, Ehud Grossman, Majdi Halabi, Ilana Harman-Boehm, Idit Liberty, Oscar Minuchin, Ofri Mosenzon, Farid Nakhoul, Assy Nimer, Doron Schwartz, Julio Wainstein, Yoram Yagil, and Robert Zukermann.

Italy: Aneliy Ilieva Parvanova, Carlo Bruno Giorda, Enzo Bonora, Paolo Calabrò, Davide Carlo Maggi, Roberto Cimino, Salvatore David, Michele Emdin, Antonio Ettore Pontiroli, Enrico Fiaccadori, Paolo Fiorina, Giovanni Giorgio Battaglia, Maria Cristina Gregorini, Gaetano La Manna, Giorgio Luciano Viviani, Franco Luigi Cavalot, Roberta Manti, Anna Maria Veronelli, Giancarla Meregalli, Antonello Pani, Norberto Perico, PierMarco Piatti, Antonio Pisani, Paola Ponzani, Gennaro Santorelli, Domenico Santoro, Renzo Scanziani, Ugo Teatini, Maurizio Tiziano Bevilacqua, Giancarlo Tonolo, and Roberto Trevisan.

Japan: Hideo Araki, Yukihiro Bando, Osamu Ebisui, Naruhiro Fujita, Hirotaka Fukasawa, Ryuichi Furuya, Yoshiyuki Hamamoto, Akihiro Hamasaki, Kotaro Hasegawa, Masahiro Hatazaki, Terumasa Hayashi, Masayuki Hayashi, Nobuyoshi Higa, Takayuki Higashi, Yoshihide Hirohata, Shuji Horinouchi, Ayumu Hoshi, Hirofumi Imoto, Akemi Inagaki, Masayuki Inagaki, Daijo Inaguma, Toshihiko Inoue, Masao Ishii, Tamayo Ishiko, Motohide Isono, Hideaki Jinnouchi, Hidetoshi Kanai, Daisuke Kanda, Hideo Kanehara,

Masayuki Kashima, Yuko Kataoka, Shigehiro Katayama, Kiyoe Kato, Takeshi Katsuki, Katsunori Kawamitsu, Daiji Kawanami, Satsuki Kawasaki, Fumi Kikuchi, Hidetoshi Kikuchi, Rui Kishimoto, Kunihisa Kobayashi, Junko Koide, Rieko Komi, Miyuki Kubota, Genpei Kuriya, Takeshi Kurose, Yoshiro Kusano, Hajime Maeda, Sunao Matsubayashi, Kazunari Matsumoto, Naoya Matsumura, Yasuto Matsuo, Naoki Matsuoka, Hiroaki Miyaoka, Satoshi Miyata, Takeshi Morita, Isao Murakami, Satoshi Murao, Udai Nakamura, Mikihiro Nakayama, Jun Nakazawa, Sakae Nohara, Takashi Nomiyama, Masayuki Noritake, Yoshiaki Oda, Takayuki Ogiwara, Hiroshi Ohashi, Hideki Okamoto, Shinichi Okino, Takeshi Osonoi, Nobuhiro Sasaki, Yoshitaka Sayo, Taiji Sekigami, Taro Shibasaki, Hirotaka Shibata, Tatsushi Shimoyama, Junji Shinoda, Hiroshi Sobajima, Kazuya Sugitatsu, Toshiyuki Sugiura, Toru Sugiyama, Daisuke Suzuki, Masaaki Suzuki, Asami Takeda, Asami Tanaka, Seiichi Tanaka, Izumi Tsunematsu, Yasuo Ueda, Soichi Uekihara, Makoto Ujihara, Ken Yajima, Daishiro Yamada, Masayo Yamada, Kazuo Yamagata, Fumiko Yamakawa, Ken Yamakawa, Yoshimitsu Yamasaki, Yuko Yambe, Taihei Yanagida, Hidekatsu Yanai, Toshihiko Yanase, and Tetsuyuki Yasuda.

Lithuania: Dovile Kriauciuniene, Jurate Lasiene, Antanas Navickas, Lina Radzeviciene, Egle Urbanaviciene, Gediminas Urbonas, and Audrone Velaviciene.

Malaysia: Norhaliza Mohd Ali, Nor Azizah Aziz, Nik Nur Fatnoon Nik Ahmad, Wan Hasnul, Rizmy Najme Khir, Li Yuan Lee, Chek Loong Loh, Masni Mohamad, Jeyakantha Ratnasingam, Tong Boon Alexander Tan, and Wan Mohd Izani Wan Mohamed.

Mexico: Melchor Alpizar Salazar, Sandro Avila Pardo, Miriam Bastidas Adrian, Alfredo Chew Wong, Jorge Escobedo de la Peña, Guillermo Fanghänel Salmón, Pedro García Hernández, José González , Guillermo González Gálvez, Ramiro Gutiérrez Ochoa, José Lazcano Soto, Magdalena Madero Rovalo, Gustavo Méndez Machado, Luis Nevarez Ruiz, Gabriel Ramos López, Arturo Saldaña Mendoza, Sergio Irizar Santana, Leobardo Sauque Reyna, Gustavo Solache Ortiz, Rafael Valdez Ortiz, Elvira González Vilchis, and Juan Villagordoa Mesa.

Netherlands: Rene Bakker, Jos Barendregt, Arnold Boonstra, Catherine Brouwer, Ron Gansevoort, Adriaan Kooy, Marielle Krekels, Aloysius Lieverse, Peter Luik, Lars Penne, Bert-Jan van den Born, Marjolijn van Buren, and Ruud van Leendert.

New Zealand: John Baker, Veronica Crawford, Rick Cutfield, Peter Dunn, Jeremy Krebs, Kingsley Nirmalaraj, Russell Scott, Nine Smuts, and Janet Titchener.

Norway: Emil Asprusten, Erik Eriksen, Trine Finnes, Robert Hagemeier, Hans Høivik, Kjetil Høye, Thomas Karlsson, Peter Scott Munk, Knut Risberg, Jan Rocke, Hilde Selsås, Leidulv Solnør, Frode Thorup, and Cecilie Wium.

Philippines: Albert Bautista, Elizabeth Catindig, Carlo Manalo, Roberto Mirasol, Glenda Pamugas, Maribel Tanque, and Louie Tirador.

Poland: Patrycja Butrymowicz, Kazimierz Ciechanowski, Grazyna Cieslik, Edward Franek, Janusz Gumprecht, Michal Hoffmann, Krystyna Jedynasty, Jolanta Krzykowska, Ilona Kurnatowska, Katarzyna Landa, Adam Madrzejewski, Katarzyna Madziarska, Stanislaw Mazur, Piotr Napora, Michal Nowicki, Anna Ocicka-Kozakiewicz, Barbara Rewerska, Teresa Rusicka, Jan Ruxer, Izabela Sein Anand, Ewa Skokowska, Andrzej Stankiewicz, Agnieszka Tiuryn-Petrulewicz, Katarzyna Wasilewska, Bogna Wierusz-Wysocka, and Renata Wnetrzak-Michalska.

Portugal: Edgar Almeida, Ana Rita Alves, Rosa Ballesteros, Carlos Barreto, Ilidio Brandao, Rui Carvalho, Joao Coelho, Cesar Esteves, Jose Guia, Susana Heitor, Ana Lourenco, Pedro Matos, Pedro Melo, Fernando Nolasco, Amalia Pereira, Cristina Roque, Vanisa Rosario, Francisco Rosario, Joao Sergio Neves, Gil Silva, Ana Silva, Fernando Teixeira e Costa, and Ana Vila Lobos.

Puerto Rico: Yudit Brito-Peguero, Gildred Colon-Vega, Gregorio Cortes-Maisonet, Gregorio Cortes-Maisonet, and Amaury Roman-Miranda.

Romania: Adrian Albota, Cornelia Bala, Hortensia Barbonta, Elena Caceaune, Ciprian Constantin, Adriana Dumitrescu, Adriana Filimon, Nicoleta Mindrescu, Cristina Mistodie, Gabriela Negrisanu, Adriana Onaca, Silvia Paveliu, Ella Pintilei,

Lavinia Pop, Amorin Popa, Alexandrina Popescu, Iosif Szilagyi, Liana Turcu, Georgeta Vacaru, Ioan Veresiu, and Adrian Vlad

Russia: Mikhail Antsiferov, Yulia Argunova, Mikhail Arkhipov, Andrey Babkin, Vitaliy Baranov, Olga Barbarash, Elena Chernyavskaya, Arkadiy Demko, Alexander Dreval, Anton Edin, Polina Ermakova, Albert Galyavich, Leyla Gaysina, Ivan Gordeev, Irina Ipatko, Marina Kalashnikova, Yuriy Khalimov, Vadim Klimontov, Zhanna Kobalava, Elena Kosmacheva, Natalya Koziolova, Lyudmila Kvitkova, Sergey Levashov, Roman Libis, Natalia Malykh, Sofya Malyutina, Vyacheslav Marasaev, Vyacheslav Mareev, Vladimir Martynenko, Imad Meray, Ashot Mkrtumyan, Galina Nechaeva, Konstantin Nikolaev, Shamil Palyutin, Nina Petunina, Leonid Pimenov, Elena Rechkova, Tatyana Rodionova, Oksana Rymar, Ruslan Sardinov, Olga Semenova, Alexander Sherenkov, Elena Smolyarchuk, Oleg Solovev, Leonid Strongin, Olga Ukhanova, Nadezhda Verlan, Svetlana Villevalde, Natalya Vorokhobina, Davyd Yakhontov, Elena Zakharova, Alsu Zalevskaya, Olga Zanozina, Elena Zhdanova, Larisa Zhukova, and Tatyana Zykova.

Singapore: Chee Fang Sum, Yong Mong Bee, and Ru San Tan.

Slovakia: Jana Babikova, Ingrid Buganova, Andrej Dzupina, Peter Minarik, Zuzana Ochodnicka, Dalibor Sosovec, and Denisa Spodniakova.

South Africa: Fayzal Ahmed, Aslam Amod, Sindeep Bhana, Larry Distiller, Shaifali Joshi, Mukesh Joshi, Deepak Lakha, Essack Mitha, Gracjan Podgorski, Brian Rayner, Mohamed Sarvan, Mary Seeber, Heidi Siebert, Mohammed Tayob, Julien Trokis, Dorothea Urbach, Dirkie Jansen van Rensburg, and Louis van Zyl.

South Korea: ChoonHee Chung, HyeSoo Chung, YouCheol Hwang, Ji Hye Huh, JunGoo Kang, ChulSik Kim, HyeSoon Kim, InJoo Kim, JaeHyeon Kim, NamHoon Kim, SinGon Kim, ByungWan Lee, WooJe Lee, Soo Lim, Young Min Cho, Jae Myung Yu, and Cheol Young Park

Spain: Hanane Bouarich, Francesca Calero, Cristina Castro, Fernando Cereto Castro, Secundino Cigarrán Guldris, Josep Cruzado Garrit, Pablo Gómez Fernández, Laura Fuentes Sánchez, Josep Galcerán, Olga González Albarrán,

Mercedes González Moya, Julio Hernández, Domingo Hernández Marrero, Meritxell Ibernón, José María Pascual Izuel, Francisco Martínez Debén, Judith Martins, Juan Mediavilla, Alfredo Michán, Julio Pascual Santos, Gonzalo Piedrola Maroto, Esteban Poch, Manuel Polaina Rusillo, Josep Redón, Carlos Sánchez Juan, Rafael Santamaría Olmo, José Julián Segura de la Morena, Daniel Seron, Alfonso Soto González, and Maribel Troya.

Sweden: Dan Curiac, Ken Eliasson, Malin Frank, Gregor Guron, Olof Hellberg, Margareta Hellgren, Hans Larnefeldt, Cornelia Lif-Tiberg, Carl-Johan Lindholm, Magnus Löndahl, Johan Månflod, Han Nguyen, Erik Rein-Hedin, Inga Soveri, Jonas Spaak, and Bengt-Olov Tengmark.

Switzerland: Stefan Bilz, Michel Burnier, Markus Laimer, Antoinette Pechère-Bertschi, Gottfried Rudofsky, Bernd Schultes, Christopher Strey, and Gregoire Wuerzner.

Taiwan: Chiz-Tzung Chang, Lee-Ming Chuang, Cheng-Chieh Hung, Ju-Ying Jiang, Chien-Te Lee, Der-Cherng Tarng, Shih-Te Tu, Mai-Szu Wu, and Ming-Ju Wu.

Thailand: Chaicharn Deerochanawong, Natapong Kosachunhanan, Chatlert Pongchaiyakul, Bancha Satirapoj, and Piyamitr Sritara.

Turkey: Ozer Badak, Murat Cayli, Necmi Eren, Ibrahim Gul, Ismail Kocyigit, Abdulbaki Kumbasar, Aytekin Oguz, Oner Ozdogan, Idris Sahin, Ibrahim Sari, Ramazan Sari, Talat Tavli, Ahmet Temizhan, Mustafa Tigen, Ugur Turk, Yavuz Yenicerioglu, Huseyin Yilmaz, and Mehmet Yilmaz.

Ukraine: Iryna Bondarets, Volodymyr Botsyurko, Viktoriia Chernikova, Oleksandra Donets, Ivan Fushtey, Mariia Grachova, Ganna Isayeva, Dmytro Kogut, Julia Komisarenko, Nonna Kravchun, Oleksandr Larin, Kateryna Malyar, Borys Mankovsky, Liliya Martynyuk, Vitaliy Maslyanko, Halyna Myshanych, Larysa Pererva, Nataliia Pertseva, Oleksandr Serhiyenko, Ivan Smirnov, Liubov Sokolova, and Maryna Vlasenko.

United Kingdom: Rudy Bilous, Cuong Dang, Andrew Johnson, Hassan Kahal, Dhanya Kalathil, Anne Kilvert, Christina Kyriakidou, Amit Mathew, Kieran McCafferty, Rasha Mukhtar, Imrozia Munsoor, Sui Phi Kon, Anton Poterajlo, Sam Rice, Pauline Swift, Arutchelvam Vijayaraman, and Yuk-ki Wong.

United States: Emaad Abdel-Rahman, Edel Abreu, Idalia Acosta, Atoya Adams, Sharon Adler, Dilawar Ajani, Slamat Ali, Radica Alicic, Amer Al-Karadsheh, Sreedhara Alla, Dale Allison, Nabil Andrawis, Ahmed Arif, Ahmed Awad, Alaa Awad, Masoud Azizad, Nader Bahri, Shweta Bansal, Steven Barag, Mark Barney, Khalid Bashir, Jose Bautista, Srinivasan Beddhu, Sabrina Benjamin, Ramin Berenji, John Bertsch, Anuj Bhargava, Jose Birriel, David Bleich, Jonathan Bornfreund, Harjeet Brar, Susan Brian, Stephen Brietzke, Cynthia Brinson, Humberto Bruschetta, Osvaldo Brusco, Anna Burgner, Robert Busch, Rafael Canadas, Maria Caramori, Jose Cardona, Jose Carpio, Christopher Case, Steven Cohen, John Cosby, Humberto Cruz, Ramprasad Dandillaya, Dalia Dawoud, Soni Dhanireddy, Jorge Diaz, Zia Din, Bradley Dixon, Ankur Doshi, Fredrick Dunn, Mahfouz El Shahawy, Mohammed El-Shahawy, Sabitha Eppanapally, John Evans, George Fadda, Umar Farooq, Joseph Fayad, Roland Fernando, Brian First, David Fitz-Patrick, John Flack, Patrick Fluck, Leon Fogelfeld, Vivian Fonseca, Juan Frias, Claude Galphin, Luis Garcia-Mayol, Archana Goel, Gary Goldstein, Edgar Gonzalez, Francisco Gonzalez-Abreu, Ashwini Gore, Kanakadurga Govindaraju, David Grant, Stephen Halpern, Maxine Hamilton, Jamal Hammoud, Stuart Handelsman, Israel Hartman, Glenn Heigerick, German Hernandez, Carlos Hernandez-Cassis, Carlos Herrera, Ali Iranmanesh, Timothy Jackson, Mahendra Jain, Kenneth Jamerson, Karen Johnson, Audrey Jones, Zeid Kayali, William Kaye, Bobby Khan, Muhammad Khan, Sourabh Kharait, Sue Kirkman, Herbert Knight, Stanley Koch, Nandini Kohli, Nelson Kopyt, Gary Korff, Wayne Kotzker, Csaba Kovesdy, Camil Kreit, Arvind Krishna, Guido Lastra, Keung Lee, Brenda Lemus, Sam Lerman, Douglas Linfert, Jorge Loredo, Dragana Lovre, Alexander Lurie, Geetha Maddukuri, Alexander Magno, Louis Maletz, Mustafa Mandviwala, Mariana Markell, Earl Martin, Ronald Mayfield, Caroline Mbogua, Dierdre McMullen, Carl Meisner, Jill Meyer, Bharat Mocherla, Manuel Montero, Moustafa Moustafa, John Murray, Salil Nadkarni, Samer Nakhle, Jesus Navarro, Nilda Neyra, Romanita Nica, Philip Nicol, Paul Norwood, Visal Numrungroad, Richard O'Donovan, Adeniyi Odugbesan, David Oliver, Suzanne Oparil, Jorge Paoli-Bruno, Rajesh Patel, Rakesh Patel, Aldo Peixoto,

Jesus Penabad, Isabel Pereira, Pablo Pergola, Karlton Pettis, Roberto Pisoni, Mirela Ponduchi, Larry Popeil, Jorge Posada, Gonzalo Quesada, Kodangudi Ramanathan, Luis Ramos-Gonez, Mandana Rastegar, Anjay Rastogi, Padmashri Rastogi, Efrain Reisin, Marc Rendell, Michael Rocco, Juan Rondon, Sylvia Rosas, Dennis Ross, Jeffrey Rothman, Prabir Roy-Chaudhury, Lance Rudolph, Yusuf Ruhullah, Gary Ruoff, Jeffrey Ryu, Mandeep Sahani, Garfield Samuels, William Sanchez, Sanjeev Saxena, David Scott, Gilberto Seco, Harvey Serota, Nauman Shahid, Michael Shanik, Santosh Sharma, Arjun Sinha, James Smelser, Mark Smith, David Smith, Kyaw Soe, Richard Solomon, Eugene Soroka, Joseph Soufer, Rosa Suarez, Bala Subramanian, Harold Szerlip, Aparna Tamirisa, Stephen Thomson, Soheila Torabi, Tuan-Huy Tran, Richard Treger, Gretel Trullenque, Thomas Turk, Guillermo Umpierrez, Martin Valdes, Shujauddin Valika, Damaris Vega, Peter Weissman, Adam Whaley-Connell, Don Williamson, Jonathan Winston, Jonathan Wise, Catherine Womack, Hala Yamout, Michael Yuryev, and Steven Zeig.

Vietnam: Lam Van Hoang, Tran Khanh, Thuy Khuong Le, Boi Ngoc Nguyen, Pham Nguyen Son, Thao Nguyen, Nguyen Minh Nui, Tran Quang Nam, Lan Phuong Tran, and Kim Chi Tran.

Plain Language Summary

Why did we do this research?

People who have all or part of their kidney removed are more likely to get kidney and heart disease and can become very unwell. A previous study combined the results of two clinical trials of patients with kidney disease and type 2 diabetes. It showed that finerenone, a medication that can help people with heart or kidney disease, helped patients to live longer, slowing down kidney damage and reducing the risk of heart disease. Finerenone also reduced the amount of protein in the urine, which can be a sign that the kidneys are not working properly. This study did not provide information about patients who have had all or part of their kidney removed; therefore, we looked to see if finerenone was useful for these patients.

What did we do?

We wanted to learn if finerenone is helpful for people who have had all or part of their kidney removed. This was done by looking at the combined results from two clinical trials of finerenone in patients with kidney disease and type 2 diabetes. In these trials, we measured protein levels in the urine of people who took finerenone and people who did not take finerenone.

What did we find?

We found that in people with kidney disease and type 2 diabetes who had all or part of their kidney removed, protein levels in urine were lower if they took finerenone than if they did not take finerenone.

What does this mean?

By lowering protein levels in the urine, finerenone might help to slow the progression of kidney disease for patients with type 2 diabetes, even if they have had all or part of a kidney removed. This means that doctors have more information available for this group of patients when it comes to making treatment decisions.

Supplemental Tables and Figures

Supplemental Table 1. *P* values for the main factors and interaction terms used in the mixed model for urine albumin-to-creatinine ratio to baseline analysis

Factors/interaction terms	Nephrectomy	No Nephrectomy
	***P* value**	***P* value**
**Factors**[a]		
Treatment	<0.001	<0.001
Region	0.30	<0.001
Albuminuria at screening	0.22	<0.001
eGFR category at screening	0.67	0.40
History of CVD	0.10	0.48
Study	0.66	0.16
Time	0.82	<0.001
Baseline value nested within eGFR category at screening	0.81	0.52
**Interactions**[b]		
Treatment*time	0.30	<0.001
Baseline value*time	0.95	<0.001
Treatment*study	0.61	0.80

[a]*P* values based on F-test of equal means between the factor levels. [b]*P* values based on F-test of interaction.

CVD, cardiovascular disease; eGFR, estimated glomerular filtration rate.

Supplemental Table 2. Analysis of covariance for ratio to baseline of urine albumin-to-creatinine ratio at month 4 (closest visit[a])

Factor	Nephrectomy			No Nephrectomy		
	Level (*n*/*N*)	LS mean ratio to baseline (95% CI)	*P* value	Level (n/N)	LS mean ratio to baseline (95% CI)	*P* value
**Treatment[b,c]**	Finerenone (53/55)	0.65 (0.55–0.76)	<0.001	Finerenone (6158/6443)	0.64 (0.62–0.65)	<0.001
	Placebo (51/53)	1.08 (0.91–1.27)		Placebo (6115/6439)	0.93 (0.92–0.95)	
**Region**	Europe (66/68)	0.85 (0.73–0.98)	0.15	Europe (5532/5794)	0.75 (0.73–0.76)	<0.001
	North America (17/18)	0.85 (0.64–1.13)		North America (1889/2031)	0.77 (0.75–0.80)	
	Asia (9/9)	0.99 (0.67–1.47)		Asia (3060/3161)	0.80 (0.78–0.82)	
	Latin America (7/8)	0.43 (0.28–0.68)		Latin America (1346/1426)	0.78 (0.75–0.82)	
	Other (5/5)	1.05 (0.62–1.78)		Other (446/470)	0.85 (0.79–0.91)	
**Albuminuria at screening**	High[d] (32/32)	0.76 (0.62–0.94)	0.87	High[d] (3601/3801)	0.80 (0.78–0.82)	0.05
	Very high[e] (72/76)	0.86 (0.75–0.99)		Very high[e] (8672/9081)	0.76 (0.75–0.77)	
**eGFR category at screening**	eGFR 25–<45 (54/56)	0.94 (0.80–1.10)	0.19	eGFR 25–<45 (4090/4312)	0.81 (0.79–0.83)	<0.001
	eGFR 45–<60 (26/27)	0.72 (0.57–0.91)		eGFR 45–<60 (3385/3553)	0.78 (0.76–0.80)	

Factor	Nephrectomy			No Nephrectomy		
	Level (*n*/*N*)	LS mean ratio to baseline (95% CI)	*P* value	Level (n/N)	LS mean ratio to baseline (95% CI)	*P* value
	eGFR ≥60 (24/25)	0.74 (0.58–0.94)		eGFR ≥60 (4798/5017)	0.73 (0.71–0.75)	
History of CVD	CVD present (48/108)	0.90 (0.76–1.07)	0.27	CVD present (5559/12,882)	0.77 (0.75–0.79)	0.36
	CVD absent (56/108)	0.77 (0.66–0.91)		CVD absent (6714/12,882)	0.77 (0.76–0.79)	
Study[b,c]	FIDELIO-DKD (57/108)	0.89 (0.76–1.04)	0.50	FIDELIO-DKD (5347/12,882)	0.79 (0.77–0.81)	0.28
	FIGARO-DKD (47/108)	0.76 (0.64–0.90)		FIGARO-DKD (6926/12,882)	0.76 (0.74–0.77)	

[a]Visit closest to day 120 within a time window of 120 ± 30 days after randomization; if no measurements were available in this time window, the patient was excluded from the analysis. [b]$P_{interaction}$ for study*treatment (nephrectomy group) = 0.86. [c]$P_{interaction}$ for study*treatment (no nephrectomy group) = 0.51. [d]High albuminuria is 30 mg/g to less than 300 mg/g. [e]Very high albuminuria is at least 300 mg/g.

Analysis of covariance with factors treatment group, region, eGFR category at screening, type of albuminuria at screening, CVD history, study, log-transformed baseline value nested within type of albuminuria, and the interaction between study and treatment.

CVD, cardiovascular disease; eGFR, estimated glomerular filtration rate; FIDELIO-DKD, Finerenone in Reducing Kidney Failure and Disease Progression in Diabetic Kidney Disease; FIGARO-DKD, Finerenone in Reducing Cardiovascular Mortality and Morbidity in Diabetic Kidney Disease; LS, least-squares; *n/N*, number of patients with UACR data available for the analysis/total number of patients in subgroup.

Supplemental Table 3. Urine albumin-to-creatinine ratio reductions up to two years according to nephrectomy status in medical history (unadjusted analysis; full analysis set)

Visit	Nephrectomy				No Nephrectomy			
	**Treatment (*n*/*N*)**	**LS mean ratio to baseline (95% CI)**	**LS mean treatment ratio (95% CI)**	***P* value**	**Treatment (*n*/*N*)**	**LS mean ratio to baseline (95% CI)**	**LS mean treatment ratio (95% CI)**	***P* value**
Month 4	Finerenone (53/55)	0.65 (0.53–0.79)	0.60 (0.47–0.76)	<0.001	Finerenone (6199/6443)	0.64 (0.62–0.65)	0.68 (0.66–0.70)	<0.001
	Placebo (51/53)	1.08 (0.95–1.24)			Placebo (6173/6439)	0.94 (0.92–0.95)		
Month 12	Finerenone (51/55)	0.52 (0.40–0.67)	0.49 (0.36–0.66)	<0.001	Finerenone (5918/6443)	0.57 (0.55–0.59)	0.60 (0.58–0.63)	<0.001
	Placebo (47/53)	1.07 (0.89–1.29)			Placebo (5911/6439)	0.94 (0.92–0.97)		
Month 24	Finerenone (37/55)	0.63 (0.43–0.91)	0.57 (0.36–0.89)	0.01	Finerenone (4811/6443)	0.59 (0.57–0.61)	0.62 (0.59–0.65)	<0.001
	Placebo (42/53)	1.11 (0.85–1.45)			Placebo (4773/6439)	0.95 (0.92–0.98)		

Mixed model with factors of treatment group, time, and treatment*time.

LS, least-squares; *n/N*, number of patients with urine albumin-to-creatinine ratio data available for the analysis/total number of patients in subgroup.

Supplemental Table 4. Summary of treatment-emergent adverse events by body system or organ class (safety analysis set)

Body system or organ class, *n* (%)	Nephrectomy		No Nephrectomy	
	Finerenone (*n*=55)	Placebo (*n*=53)	Finerenone (*n*=6434)	Placebo (*n*=6421)
Blood and lymphatic system disorders	4 (7)	4 (8)	676 (11)	671 (10
Cardiac disorders	8 (15)	7 (13)	793 (12)	908 (14)
Congenital, familial and genetic disorders	0	0	33 (0.5)	40 (0.6)
Ear and labyrinth disorders	0	1 (2)	153 (2)	150 (2)
Endocrine disorders	5 (9)	2 (4)	168 (3)	181 (3)
Eye disorders	11 (20)	9 (17)	901 (14)	966 (15)
Gastrointestinal disorders	14 (25)	13 (25)	1807 (28)	1764 (27)
General disorders and administration site conditions	13 (24)	5 (9)	1406 (22)	1617 (25)
Hepatobiliary disorders	2 (4)	1 (2)	457 (7)	463 (7)
Immune system disorders	1 (2)	0	145 (2)	123 (2)
Infections and infestations	25 (45)	25 (47)	2714 (42)	2763 (43)
Injury, poisoning, and procedural complications	11 (20)	14 (26)	983 (15)	952 (15)
Investigations	3 (5)	1 (2)	480 (7)	520 (8)
Metabolism and nutrition disorders	13 (24)	12 (23)	2443 (38)	2152 (34)
Musculoskeletal and connective tissue disorders	20 (36)	15 (28)	1922 (30)	1978 (31)
Neoplasms benign, malignant, and unspecified (incl. cysts and polyps)	9 (16)	7 (13)	476 (7)	491 (8)
Nervous system disorders	7 (13)	9 (17)	1383 (21)	1388 (22)
Pregnancy, puerperium, and perinatal conditions	0	0	0	1 (<0.1)
Product issues	0	0	8 (0.1)	11 (0.2)
Psychiatric disorders	4 (7)	3 (6)	381 (6)	448 (7)
Renal and urinary disorders	11 (20)	15 (28)	1410 (22)	1414 (22)
Reproductive system and breast disorders	3 (5)	3 (6)	345 (5)	350 (5)

Body system or organ class, *n* (%)	Nephrectomy		No Nephrectomy	
	**Finerenone (*n*=55)**	**Placebo (*n*=53)**	**Finerenone (*n*=6434)**	**Placebo (*n*=6421)**
Breast hyperplasia	0	0	0	5 (<0.1)
Gynecomastia	0	0	8 (0.1)	11 (0.2)
Respiratory, thoracic, and mediastinal disorders	11 (20)	14 (26)	1177 (18)	1225 (19)
Skin and subcutaneous tissue disorders	5 (9)	5 (9)	837 (13)	855 (13)
Social circumstances	0	0	2 (<0.1)	4 (<0.1)
Surgical and medical procedures	6 (11)	7 (13)	350 (5)	306 (5)
Vascular disorders	10 (18)	12 (23)	1241 (19)	1285 (20)

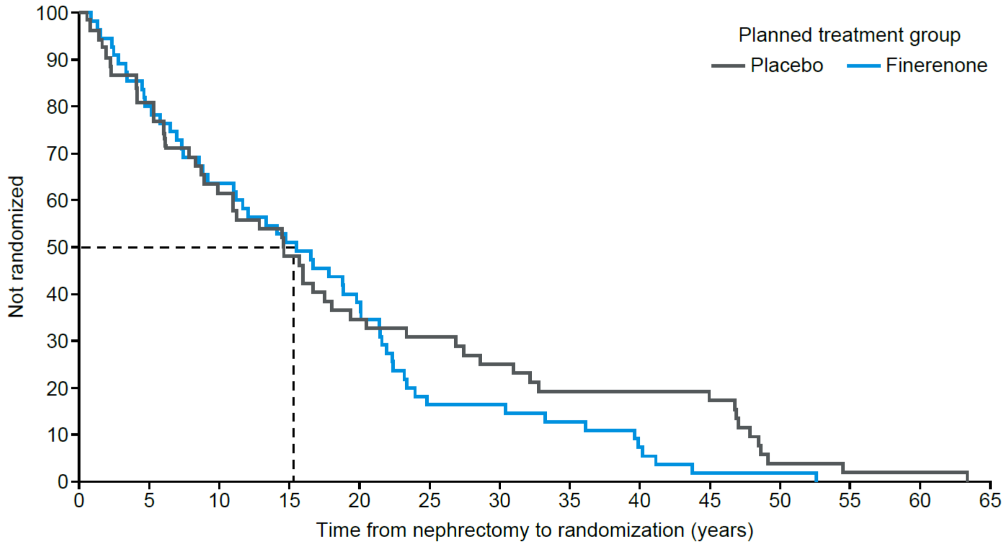

No. of patients not randomized

Placebo	52	42	32	25	18	16	13	10	10	9	2	1	1	0
Finerenone	55	44	35	28	21	9	9	7	4	1	1	0	0	0

Supplemental Figure 1. Time from nephrectomy to randomization.

Number of patients not randomized was considered at the start of the time point. Partial missing nephrectomy start date was imputed by the median rule (missing day only was imputed by the 15th of the month, missing day and month were imputed by July 1). Patients with completely missing start dates were not included in the analysis.
